# Impact of an Educational Intervention on the Opioid Knowledge and Prescribing Behaviors of Resident Physicians

**DOI:** 10.7759/cureus.23508

**Published:** 2022-03-26

**Authors:** Pankti P Acharya, Brianna R Fram, Jenna R Adalbert, Ashima Oza, Prashanth Palvannan, Evan Nardone, Nicole Caltabiano, Jennifer Liao, Asif M Ilyas

**Affiliations:** 1 Pain Management, Rowan University School of Osteopathic Medicine, Stratford, USA; 2 Orthopaedic Surgery, Thomas Jefferson University, Philadelphia, USA; 3 Orthopaedic Surgery, Sidney Kimmel Medical College, Philadelphia, USA; 4 Internal Medicine, Thomas Jefferson University Hospital, Philadelphia, USA; 5 General Surgery, Thomas Jefferson University Hospital, Philadelphia, USA; 6 Emergency Medicine, Thomas Jefferson University Hospital, Philadelphia, USA; 7 Rothman Opioid Foundation, Rothman Orthopaedic Institute at Thomas Jefferson University, Philadelphia, USA

**Keywords:** pain management, resident physicians, educational intervention, opioid epidemic, opioid education

## Abstract

Objectives: The opioid epidemic is a multifactorial issue, which includes pain mismanagement. Resident physician education is essential in addressing this issue. We aimed to analyze the effects of an educational intervention on the knowledge and potential prescribing habits of emergency medicine (EM), general surgery (GS), and internal medicine residents (IM).

Methods: Resident physicians were provided with educational materials and were given pre-tests and post-tests to complete. Descriptive statistics were used to analyze pre-test and post-test responses. Chi-squared analysis was used to identify changes between the pre-tests and post-tests. A p < 0.05 value was considered statistically significant.

Results: Following the educational intervention, we observed improvement in correct prescribing habits for acute migraine management among emergency medicine residents (from 14.8% to 38.5%). Among general surgery residents, there was a significant improvement in adherence to narcotic amounts determined by recent studies for sleeve gastrectomy (p= 0.01) and laparoscopic cholecystectomy (p= 0.002). Additionally, we observed a decrease in the number of residents who would use opioids as a first-line treatment for migraines, arthritic joint pain, and nephrolithiasis.

Discussion: Resident physicians have an essential role in combating the opioid epidemic. There was a significant improvement in various aspects of opioid-related pain management among emergency medicine, internal medicine, and general surgery residents following the educational interventions. We recommend that medical school and residency programs consider including opioid-related pain management in their curricula.

## Introduction

The United States (US) opioid epidemic is a multifactorial crisis, with prescription opioids identified as a key contributor to opioid misuse and overdose deaths [1]. At the medical provider level, harm reduction techniques have focused on preventing an excess of prescription opioids from circulating in the community. Approaches to this have included legislative limits on prescription amounts for certain patient populations and state-mandated use of prescription drug monitoring programs (PDMPs) to regulate opioid dispensing [2-3]. While recent studies have provided specialty- and procedure-specific opioid prescribing recommendations based on patient consumption patterns and pain relief requirements, no formal prescribing guidelines exist to eradicate the provider uncertainty that stems from the fear of undermanaging patient pain [4-7]. Additionally, medical school and residency program curricula dedicated to key opioid and pain management topics are underwhelming in the context of the severity of the opioid epidemic. This has been attributed partly to a limited pool of faculty who feel qualified to teach these concepts and to a lack of standardized competencies driving curricular design [7,8].

 Accordingly, residents across all medical disciplines are often underprepared to prescribe opioids for patient pain or respond to various opioid-related patient management scenarios [9-11]. The magnitude of this deficit is well-exemplified in a recent study surveying surgical residents at a large academic institution: 90% reported no formal training in best practices of pain management or opioid prescribing, despite reliance on opioids for postoperative pain management [11]. In response to this insufficiency in medical trainee preparation, residency programs have begun to incorporate opioid and pain management material into their curricula. Programs have used various educational models, and some have quantified the effectiveness of these didactics through methods such as survey data collection [11-13]. However, these interventions are typically implemented in a specialty-specific cohort, which limits group knowledge comparisons and the potential to evaluate standardized intervention effectiveness across a variety of medical disciplines. Raheemullah et al. conducted an opioid education intervention using pre-tests and post-tests among internal medicine residents and found improvement in knowledge and prescribing habits [14].

The purpose of this study was to investigate the impact of an educational presentation on resident knowledge and attitudes related to opioid prescribing and pain management, in internal medicine (IM), general surgery (GS), and emergency medicine (EM) residents. By implementing a standardized intervention designed to educate trainees on key concepts such as opioid crisis statistics, opioid prescribing laws, opioid-related complications, and evidence-based opioid prescribing guidelines, we aimed to measure the success of this intervention at content delivery while simultaneously collecting data on the opioid and pain management education of our residents. The goal was to compare the effectiveness of this educational model at improving resident opioid and pain management knowledge, attitudes, and behaviors across several specialties and assess the feasibility of a generalized institutional approach to resident opioid education.

This article was previously posted on Research Square as a Preprint on August 17, 2021.

## Materials and methods

This study was determined to be exempt from institutional review board review by the Review Board of Thomas Jefferson University. A total of 46 IM, 17 GS, and 27 EM residents from all postgraduate years (PGYs) at Thomas Jefferson University Hospital in Philadelphia, Pennsylvania, were recruited by email to voluntarily participate in this study. The intervention was designed as a seven-minute pre-recorded lecture with accompanying pre-tests and post-tests. Tests were intended to assess resident opioid and pain management knowledge, attitudes, and behaviors at baseline and upon presentation completion. Lecture content consisted of opioid crisis statistics, opioid prescribing laws, opioid-related complications, and evidence-based opioid prescribing guidelines with practice recommendations modified for each specialty. The pre-tests and post-tests were designed by a team of physicians and medical students. Each test was identical for each group of residents, with differences only in case vignette content and prescribing guidelines between the three cohorts to provide residents with recommendations and scenarios relevant to their specific fields (see Appendix). The post-tests were taken shortly after the pre-tests. The complete pre-tests and post-tests for each specialty are available in the Appendix. The data from the pre-tests and post-tests were organized and we performed descriptive statistics to quantify the responses. We used a Chi-squared analysis to identify resident changes between baseline and completion of the educational intervention using IBM SPSS Statistics for Windows, Version 26.0 (Released 2019; IBM Corp, Armonk, New York). Additionally, we generated comparisons of performance measures across the three cohorts to identify specialty-specific trends. A p < 0.05 value was considered statistically significant. 

## Results

A total of 90 residents completed the pre-tests; there were 27 residents from EM, 17 from GS, and 46 from IM. There were 46 post-test responses from 13 EM residents, 13 GS, and 20 IM residents. The response rates between pre-test and post-test for EM, GS, and IM were 48%, 76%, and 43% respectively. The demographics for the residents are listed in Table 1.

**Table 1:** Resident demographics PGY: post-graduate year; DEA: Drug Enforcement Administration

Emergency Medicine			
	Pre-test	Post-test	
Year in Residency			
PGY1	8 (30%)	5 (38%)	
PGY2	8 (30%)	2 (15%)	
PGY3	11 (41%)	6 (46%)	
DEA License			
Yes	0 (0%)	0 (0%)	
No	27 (100%)	27 (100%)	
General Surgery			
	Pre-test	Post-test	
Year in Residency			
PGY1	2 (12%)	0 (0%)	
PGY2	2 (12%)	3 (23%)	
PGY3	3 (18%)	3 (23%)	
PGY4	5 (29%)	4 (31%)	
PGY5	4 (24%)	3 (23%)	
DEA License			
Yes	15 (88%)	13 (100%)	
No	2 (12%)	0 (0%)	
Internal Medicine			
	Pre-test	Post-test	
Year in Residency			
PGY1	18 (39%)	9 (45%)	
PGY 2	12 (26%)	8 (40%)	
PGY3	16 (35%)	3 (15%)	
DEA License			
Yes	4 (9%)	0 (0%)	
No	42 (91%)	20 (100%)	

EM residents reported receiving education about opioids from various avenues and stages of training, including personal reading (10 residents (37%)), medical school (16 residents (59.3%)), and residency (22 residents (81.5%)). Regarding training previously received, four residents were very satisfied (14.8%), eight were satisfied (29.6%), 11 were neutral (40.7%), three were unsatisfied (11.1%), and one was very unsatisfied (3.7%). The EM resident prescribing habits and opioid knowledge are listed in Table 2. After receiving the educational intervention, the attitudes of EM residents to the statement “If I suspect someone is abusing opioids, I do not prescribe opioids to them” significantly changed (p=0.04).

**Table 2:** EM resident knowledge and attitudes NSAID: non-steroidal anti-inflammatory drug

	Pre-test	Post-test	P-value
For an adult patient that presents to the emergency room with acute pain, according to current PA state guidelines, what is the maximum duration (days) for which an opioid prescription should be given?			
7 days	6 (22.2%)	4 (30.8%)	0.56
For an adult presenting to the ED with acute low back pain, I would typically prescribe:			
0-10 tablets of 5mg oxycodone + NSAID	0 (0%)	2 (15.4%)	0.54
A 25-year-old female presents to the office with an acute episodic migraine According to the American Headache Society 2015 Guidelines, what treatment has Level A evidence?			
Naratriptan	4 (14.8%)	5 (38.5%)	0.09
I feel comfortable in my knowledge of non-opioid pain management.			
Agree	15 (55.5%)	7 (53.8%)	0.06
Strongly agree	5 (18.5%)	3 (23.1%)	
If I suspect someone is abusing opioids, I do not prescribe opioids to them.			
Agree	12 (44.4%)	2 (15.4%)	0.04
Strongly agree	7 (25.9%)	2 (15.4%)	
For patients experiencing moderate pain, I usually initially prescribe:			
Tylenol	5 (18.5%)	5 (38.5%)	0.17
NSAIDs	22 (81.5%)	8 (61.5%)	
Opioid	0 (0%)	0 (0%)	

Comparatively, GS residents received opioid training from personal reading (five residents, 29%) medical school (nine residents, 53%), and residency (17 residents, 100%). Regarding prior opioid training, one resident felt unsatisfied, four residents felt neutral, nine residents felt satisfied (52.9%), and three felt very satisfied (17.6%). Following this educational intervention, one resident felt unsatisfied (7.7%), seven felt satisfied (53.8%), and five felt very satisfied (38.5%) with their opioid-prescribing abilities. Furthermore, there was a significant improvement in prescribing knowledge following a sleeve gastrectomy (p=0.01) and a laparoscopic cholecystectomy (p=0.002). The GS resident prescribing habits are listed in Table 3.

**Table 3:** General surgery specific questions *= correct answer

		Correct responses (%)	Incorrect responses (%)				
For a patient being discharged home after a sleeve gastrectomy, I would typically prescribe:							
0-10 tablets 5mg oxycodone*	9 (52.9%)			13 (100%)		0.01*	
For a patient being discharged home after a laparoscopic cholecystectomy, I would typically prescribe:							
0-15 tablets*	1 (5.9%)			7 (53.8%)		0.002*	
For a patient being discharged home after an open small bowel resection, I would typically prescribe:							
0-15 tablets*	6 (35.3%)			8 (61.5%)		0.16	
For a patient being discharged home after a major hernia repair, I would typically prescribe							
0-10 tablets*	4 (23.5%)			9 (69.2%)		0.04*	

The IM residents reported receiving opioid training from personal reading (14, 30.4%), medical school (28, 60.9%), residency (33, 71.7%), or in some cases, never received training (4, 8.7%). Regarding their previous opioid training, one resident was very unsatisfied (4.3%), 21 residents (45.7%) were unsatisfied, 19 (41.3%) were neutral, three (6.5%) were satisfied, and two were very satisfied (4.3%). Following the study training, nine residents were unsatisfied (45%), nine were neutral (45%), and two were satisfied (10%). Following the educational intervention, there was an improvement in responses to multiple treatment scenarios, though none of this reached significance. These included treatment of acute episodic migraines according to American Headache Society 2015 Guidelines (45.7% to 70% prescribing naratriptan, p=0.11), improvement in prescribing habits for joint pain in a patient with a history of osteoarthritis (23.9% to 45%, p=0.14), and an increase in non-opioid management of nephrolithiasis in a patient with no history of GI bleed (62.2% to 70%, p=0.59) (Table 4).

**Table 4:** Internal medicine specific questions *= correct answer

If I suspect someone is abusing opioids, I do not prescribe opioids to them.			
Agree	25 (54.3%)	9 (45%)	0.22
Strongly Agree	8 (17.4%)	9 (45%)	
I think that proper pain management is associated with better patient outcomes.			
Agree	25 (54.3%)	14 (70%)	0.11
Strongly Agree	20 (43.5%)	6 (30%)	
A 25-year-old female presents to the office with an acute episodic migraine According to the American Headache Society 2015 Guidelines, what treatment has Level A evidence?			
Naratriptan*	21 (45.7%)	14 (70%)	0.11
A 65-year-old man returns to the clinic for joint pain in his knees. He has a history of osteoarthritis and states that it is difficult for him to complete daily tasks. His pain was not treated by NSAIDs or weight loss. What should be the next line of treatment?			
Tramadol*	11 (23.9%)	9 (45%)	0.14

**Figure 1:**
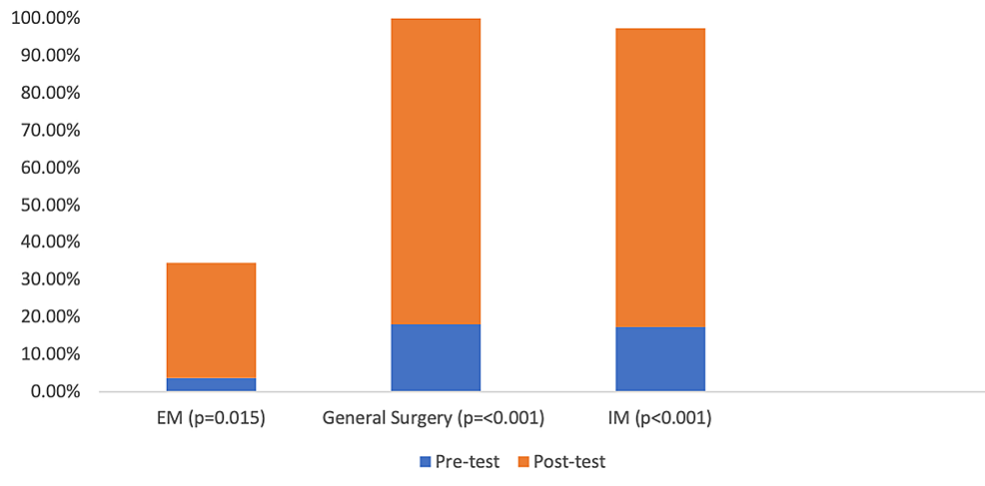
Correct responses to the number of deaths due to heroin overdose in 2017 EM: emergency medicine; IM: internal medicine

All three groups of residents were asked questions about opioid background knowledge and attitudes. In response to “Which three states have the highest percentage of opioid-related deaths per capita?”, there was a significant improvement in GS (p=0.001) and IM (p=0.003) responses following the intervention. Furthermore, there was an increase in knowledge of the number of drug overdose deaths that occurred from opioids, though it did not reach statistical significance, in both GS (41% to 77%, p=0.07) and IM (45.7% to 65%, p=0.15). Lastly, there was a significant improvement in all specialties regarding knowledge of the number of deaths that were a result of heroin overdose (GS p<0.001, IM p<0.001, EM p=0.015) (Figure 1).

Regarding the level of satisfaction with prior opioid training, there was a significant difference between specialties (p<0.0001). Almost half of all IM residents felt unsatisfied with their prior opioid training (unsatisfied or very unsatisfied - 47.9%). Comparatively, 5.9% of GS residents and 14.8% of EM residents felt unsatisfied or very unsatisfied with their training. There was also a significant difference across specialties in the initial management of mild pain (p=0.005) and moderate pain (p<0.001). For moderate pain, GS residents (35.5%) were more likely to prescribe opioids than their colleagues in IM (2.2%) and EM (0%).

## Discussion

The opioid epidemic in the US has progressively worsened. There are several historical factors that contributed to the rise of the opioid epidemic, including the classification of pain management as a human right [15] and a fifth vital sign [16], pharmaceutical marketing [17], and postoperative pain mismanagement [18]. To combat the epidemic, there has been growth in non-opioid treatments in pain management, such as nerve blocks, non-steroidal anti-inflammatory drugs (NSAIDs), and ketamine [19]. This study aimed to assess the baseline responses and the effect of a brief educational intervention on the knowledge and attitudes of EM, GS, and IM residents at a single institution. 

This study found many significant opportunities for improvement in resident opioid education. Regarding previous opioid knowledge training, 45.7% of IM residents were unsatisfied with the quality of training they had received. This suggests an area of potential collaboration between residents and hospital administration to better equip trainees with the practical information and skills they need to safely and effectively manage pain. 

With this brief intervention, there was an improvement of prescribing habits across all specialties. In EM, we observed a greater percentage of residents indicating knowledge that, per Pennsylvania state guidelines, seven days is the maximum duration of opioids that should be prescribed to an adult patient presenting to the ED with acute pain (22.2% to 30.8%). Additionally, there was an increase in the correct use of naratriptan for acute migraine management in the ED (from 14.8% to 38.5%). This particular scenario represents a key opportunity to reduce opioid use in exchange for a more efficacious medication. A study conducted by Colman et al. found that more than half of all patients presenting with migraines were treated with opioids as first-line therapy across four different hospitals [20]. Focusing on common clinical presentations like this, where treatment algorithms may be ambiguous for many providers, could greatly reduce the unnecessary use of opioids. Additionally, this effort is not meant to create a divide between providers and patients. Patients who have a history of drug misuse should receive the appropriate pharmacotherapy and psychosocial counseling to equip them with the tools to make effective change [21].

From the provider perspective, it is imperative to keep the patient’s best interest in mind when treating someone struggling with drug dependence, without letting biases affect your judgement. We observed a change in perspective among EM residents. Initially, majority of residents would not prescribe opioids to someone who appeared to be misusing drugs (70.3%). After our intervention, the number of residents who agreed with this statement decreased to 30.8% (p=0.04). This change in perspective highlights the multifaceted and individualized approach needed for each patient, considering the dangers of both over and under-prescribing. Walter et al. observed significant improvement in knowledge and management of opioid use disorder among EM residents following an educational intervention [22].

In our study, we observed significant improvement for GS in prescribing habits, better conforming to narcotic amounts determined by recent papers, following common procedures such as sleeve gastrectomy (p= 0.01), and laparoscopic cholecystectomy (p= 0.002) [23,24]. A similar study conducted by Hill et al. found that an educational intervention effectively decreased the number of opioids prescribed to patients following general surgery procedures [25].

Among IM residents, there was a decrease in participants who wanted to use opioids as a first-line treatment for migraines, arthritic joint pain, and nephrolithiasis. While these findings may not reach statistical significance, the increased percentage of correct responses indicates improvement of knowledge. The recommended first-line treatment for acute migraine includes NSAIDs and triptans. Opioid use in migraine treatment has not shown to have significant improvement so they are not recommended as initial treatment [26]; however, studies such as Bigal et al. have found that opioids were commonly used in clinical practice for migraine treatment (20.8%) [27]. A possible solution in this gap between recommendations and clinical practice can be educational interventions such as this study to target specific clinical situations that are confusing for providers or commonly treated inappropriately with opioids when good alternatives exist.

Potential limitations in this study can be attributed to the study design. Since our study focused on survey responses, the data largely depended on completion of both pre-tests and post-tests. There was a discrepancy in response rates between the two tests, likely due to survey fatigue and the demands of residents’ schedules. Additionally, this study took place during the coronavirus disease 2019 (COVID-19) pandemic, which placed considerable stress on resident physicians [28]. In order to boost survey responses, we sent reminders via email, had participating residents from each department make announcements at weekly meetings, and sent other team members to attend departmental conferences. Despite our best efforts, however, we were unable to improve these response rates. Additionally, our data is from survey answers and not real-world clinical actions. Given residents are largely constrained in their medication prescribing practices by the desires and preferences of supervising attendings, we did not feel studying their prescribing behaviors would yield meaningful results. Resident physicians are an integral component in battling the opioid epidemic. With these findings, we encourage medical schools and residency programs to integrate training on the effective use of non-opioid pain treatments into their curricula.

## Conclusions

The opioid epidemic is a multifaceted issue that can be attributed to many causes. Resident physicians are a key resource in combating the opioid epidemic. We observed significant improvement in opioid knowledge and prescribing habits among all residents following the specialty-specific educational interventions. Therefore, we recommend that medical school and residency programs consider integrating opioid-related pain management strategies throughout their curricula.

## Appendices

**Table 5:** Emergency medicine pre-test PGY: post-graduate year; DEA: Drug Enforcement Administration; PDMP: prescription drug monitoring program; PA: Pennsylvania; NSAID: non-steroidal anti-inflammatory drug

Emergency medicine resident knowledge and attitudes pre-test
Resident background
Please select your current level of training:
PGY1
PGY2
PGY3
Do you hold a DEA License?
Yes
No
How satisfied are you with your current level of opioid-prescribing training?
Very satisfied
Satisfied
Neutral
Unsatisfied
Very unsatisfied
When did you receive your opioid-prescribing training? (Select all that apply)
College
Medical School
Residency
Personal reading
Never received any formal training
For the following questions, answer as if you are the prescriber even if you do not currently hold a DEA license. Select only ONE answer unless specified otherwise.
General opioid knowledge
What are the three most common chief complaints for adults in the ED that were discharged with opioids? (select three)
Headache
Dental pain
Chest pain
Abdominal pain
Urolithiasis
Back pain
Which three states have the highest percentage of opioid-related deaths per capita: (circle 3 states)
Alabama
California
Kentucky
New York
Ohio
Pennsylvania
South Carolina
West Virginia
In 2017, how many drug overdose deaths were due to opioids?
15,000
25,000
45,000
75,000
In 2017, how many deaths were a result of heroin overdose?
15,000
25,000
45,000
75,000
Nearly half of all opioid related overdoses are due to valid prescription opioids.
True
False
What is the PDMP?
Physician Drug Medical Plan
Prescribing Directory of Medical Providers
Prescription Drug Monitoring Program
Planned Drug Movement Plan
How often should the PDMP be referenced?
Once a day
Once a month
Once a year
Anytime an opioid prescription is given.
Case-based scenarios
For an adult patient that presents to the emergency room with acute pain, according to current PA state guidelines, what is the maximum duration (days) for which an opioid prescription should be given?
0 day
1 day
3 days
7 days
14 days
No limit
When prescribing opioids to a minor, according to current PA state guidelines, the provider should:
Discuss possible risks with both the minor and parent/guardian
Document if the patient is an emancipated minor
Document the consent given
All of the above
For an adult presenting with noncancer pain, what should be the first course of action prior to formulating a pain control plan? (circle only one)
Only non-opioid pain medications
Short acting opioids
Consult the state monitoring program (PDMP)
Extended-released schedule II products
For an adult presenting to the ED with acute low back pain, I would typically prescribe: (circle only one)
Only non-opioid pain medications
0-10 tablets of 5mg oxycodone + NSAID
11-20 tablets of 5mg oxycodone + NSAID
21-30 tablets of 5mg oxycodone + NSAID
31-40 tablets of 5mg oxycodone + NSAID
41-50 tablets of 5mg oxycodone + NSAID
Over 50 tablets of 5mg oxycodone
A 25-year-old female presents to the office with an acute episodic migraine According to the American Headache Society 2015 Guidelines, what treatment has Level A evidence?
Chlorpromazine IV 12.5 mg
Celecoxib 400 mg
Codeine/acetaminophen 25/400 mg
Naratriptan 2.5 mg
Codeine 30 mg
A 30-year-old male who actively uses IV heroin presents to the ED for a localized skin infection. After several hours, he begins to complain of anxiety and GI upset. You suspect opioid withdrawal and calculate his Clinical Opiate Withdrawal Score (COWS), which at 30 is rated “moderately severe”. How would you treat his current withdrawal symptoms?
NSAIDs
Buprenorphine-naloxone to bridge him to outpatient treatment
Oral morphine
Extended-release oxycodone
Tylenol
For patients experiencing mild pain, I initially prescribe (circle one)
NSAIDs
Tylenol
Opioid
For patients experiencing moderate pain, I initially prescribe
(circle one)
NSAIDs
Tylenol
Opioid
For patients experiencing severe pain, I initially prescribe
(circle only one)
NSAID
Tylenol
Opioid
Resident attitudes
Opioids are effective in pain management.
Strongly agree
Agree
Undecided
Disagree
Strongly disagree
Every patient that presents to the ED with pain should receive opioids.
Strongly agree
Agree
Undecided
Disagree
Strongly disagree
I feel comfortable in my knowledge of non-opioid pain management.
Strongly agree
Agree
Undecided
Disagree
Strongly disagree
If I suspect someone is abusing drugs, I will not prescribe them short-acting opioids.
Strongly agree
Agree
Undecided
Disagree
Strongly disagree
Patient gender may affect my judgement of a patient's pain intensity
Strongly agree
Agree
Undecided
Disagree
Strongly disagree
Patient race may affect my judgement of a patient's pain intensity
Strongly agree
Agree
Undecided
Disagree
Strongly disagree
If a patient presents to the ED repeatedly asking for more pain medication, this could be due to a missed diagnosis of the underlying pain source.
Strongly agree
Agree
Undecided
Disagree
Strongly disagree
I ask my patients about the severity of their pain.
Strongly agree
Agree
Undecided
Disagree
Strongly disagree
I include patient-reported pain levels in my notes.
Strongly agree
Agree
Undecided
Disagree
Strongly disagree

**Table 6:** General surgery pre-test PGY: post-graduate year; DEA: Drug Enforcement Administration; PDMP: prescription drug monitoring program; PA: Pennsylvania; NSAID: non-steroidal anti-inflammatory drug

General surgery resident knowledge and attitudes pre-test
Resident background
Please select your current level of training:
PGY1
PGY2
PGY3
PGY4
PGY5
Do you hold a DEA License?
Yes
No
How satisfied are you with your current level of opioid-prescribing training?
Very satisfied
Satisfied
Neutral
Unsatisfied
Very unsatisfied
When did you receive your opioid-prescribing training? (Select all that apply)
College
Medical School
Residency
Personal reading
Never received any formal training
For the following questions, answer as if you are the prescriber even if you do not currently hold a DEA license. Select only ONE answer unless specified otherwise.
General opioid knowledge
Which three states have the highest percentage of opioid-related deaths per capita: (circle 3 states)
Alabama
California
Kentucky
New York
Ohio
Pennsylvania
South Carolina
West Virginia
In 2017, how many drug overdose deaths were due to opioids?
15,000
25,000
45,000
75,000
In 2017, how many deaths were a result of heroin overdose?
15,000
25,000
45,000
75,000
Nearly half of all opioid related overdoses are due to valid prescription opioids.
True False
What is the PDMP?
Physician Drug Medical Plan
Prescribing Directory of Medical Providers
Prescription Drug Monitoring Program
Planned Drug Movement Plan
How often should the PDMP be referenced?
Once a day
Once a month
Once a year
Anytime an opioid prescription is given
Resident attitudes
Opioids are effective in pain management.
Strongly agree
Agree
Undecided
Disagree
Strongly disagree
Every patient should receive opioids following surgery.
Strongly agree
Agree
Undecided
Disagree
Strongly disagree
I feel comfortable in my knowledge of non-opioid pain management.
Strongly agree
Agree
Undecided
Disagree
Strongly disagree
If I suspect someone is abusing opioids, I do not prescribe opioids to them.
Strongly agree
Agree
Undecided
Disagree
Strongly disagree
Patient gender may affect my judgement of a patient's pain intensity
Strongly agree
Agree
Undecided
Disagree
Strongly disagree
Patient race may affect my judgement of a patient's pain intensity
Strongly agree
Agree
Undecided
Disagree
Strongly disagree
I ask my patients about the severity of their pain.
Strongly agree
Agree
Undecided
Disagree
Strongly disagree
I include patient-reported pain levels in my notes.
Strongly agree
Agree
Undecided
Disagree
Strongly disagree
Case-based scenarios
For a patient being discharged home after an open appendectomy, I would typically prescribe: (circle only one)
Only non-opioid pain medications
0-10 tablets of 5mg Oxycodone
11-20 tablets of 5mg Oxycodone
21-30 tablets of 5mg Oxycodone
31-40 tablets of 5mg Oxycodone
41-50 tablets of 5mg Oxycodone
Over 50 tablets of 5mg Oxycodone
For a patient being discharged home after a sleeve gastrectomy, I would typically prescribe: (circle only one)
Only non-opioid pain medications
0-10 tablets of 5mg Oxycodone
11-20 tablets of 5mg Oxycodone
21-30 tablets of 5mg Oxycodone
31-40 tablets of 5mg Oxycodone
41-50 tablets of 5mg Oxycodone
Over 50 tablets of 5mg Oxycodone
For a patient being discharged home after a laparoscopic cholecystectomy, I would typically prescribe: (circle only one)
0-5 tablets of 5mg Oxycodone
0-10 tablets of 5mg Oxycodone
0-15 tablets of 5mg Oxycodone
0-20 tablets of 5mg Oxycodone
0-25 tablets of 5mg Oxycodone
Over 25 tablets of 5mg Oxycodone
For a patient being discharged home after a laparoscopic Nissen fundoplication, I would typically prescribe: (circle only one)
0-5 tablets of 5mg Oxycodone
0-10 tablets of 5mg Oxycodone
0-15 tablets of 5mg Oxycodone
0-20 tablets of 5mg Oxycodone
0-25 tablets of 5mg Oxycodone
Over 25 tablets of 5mg Oxycodone
For a patient being discharged home after an open small bowel resection, I would typically prescribe: (circle only one)
0-5 tablets of 5mg Oxycodone
0-10 tablets of 5mg Oxycodone
0-15 tablets of 5mg Oxycodone
0-20 tablets of 5mg Oxycodone
0-25 tablets of 5mg Oxycodone
Over 25 tablets of 5mg Oxycodone
For a patient being discharged home after an open colectomy, I would typically prescribe: (circle only one)
0-5 tablets of 5mg Oxycodone
0-10 tablets of 5mg Oxycodone
0-15 tablets of 5mg Oxycodone
0-20 tablets of 5mg Oxycodone
0-25 tablets of 5mg Oxycodone
Over 25 tablets of 5mg Oxycodone
For a patient being discharged home after a major hernia repair, I would typically prescribe: (circle only one)
0-5 tablets of 5mg Oxycodone
0-10 tablets of 5mg Oxycodone
0-15 tablets of 5mg Oxycodone
0-20 tablets of 5mg Oxycodone
0-25 tablets of 5mg Oxycodone
Over 25 tablets of 5mg Oxycodone
For patients experiencing mild pain, I usually initially prescribe (circle one)
NSAIDs
Tylenol
Opioid
For patients experiencing moderate pain, I usually initially prescribe
(circle one)
NSAIDs
Tylenol
Opioid
For patients experiencing severe pain, I usually initially prescribe
(circle only one)
NSAID
Tylenol
Opioid

**Table 7:** Internal medicine pre-test PGY: post-graduate year; DEA: Drug Enforcement Administration; PDMP: prescription drug monitoring program; PA: Pennsylvania; NSAID: non-steroidal anti-inflammatory drug

Internal medicine resident knowledge and attitudes pre-test
Resident background
Please select your current level of training:
PGY1
PGY2
PGY3
Do you hold a DEA License?
Yes
No
How satisfied are you with your current level of opioid-prescribing training?
Very satisfied
Satisfied
Neutral
Unsatisfied
Very unsatisfied
When did you receive your opioid-prescribing training? (Select all that apply)
College
Medical School
Residency
Personal reading
Never received any formal training
For the following questions, answer as if you are the prescriber even if you do not currently hold a DEA license. Select only ONE answer unless specified otherwise.
General opioid knowledge
Which three states have the highest percentage of opioid-related deaths per capita: (circle 3 states)
Alabama
California
Kentucky
New York
Ohio
Pennsylvania
South Carolina
West Virginia
In 2017, how many drug overdose deaths were due to opioids?
15,000
25,000
45,000
75,000
In 2017, how many deaths were a result of heroin overdose?
15,000
25,000
45,000
75,000
Nearly half of all opioid related overdoses are due to valid prescription opioids.
True False
What is the PDMP?
Physician Drug Medical Plan
Prescribing Directory of Medical Providers
Prescription Drug Monitoring Program
Planned Drug Movement Plan
How often should the PDMP be referenced?
Once a day
Once a month
Once a year
Anytime an opioid prescription is given.
Resident attitudes
Opioids are effective in pain management.
Strongly agree
Agree
Undecided
Disagree
Strongly disagree
Every patient that presents to the office with pain should receive opioids.
Strongly agree
Agree
Undecided
Disagree
Strongly disagree
I feel comfortable in my knowledge of non-opioid pain management.
Strongly agree
Agree
Undecided
Disagree
Strongly disagree
If I suspect someone is abusing opioids, I do not prescribe opioids to them.
Strongly agree
Agree
Undecided
Disagree
Strongly disagree
Patient gender may affect my judgement of a patient's pain intensity
Strongly agree
Agree
Undecided
Disagree
Strongly disagree
Patient race may affect my judgement of a patient's pain intensity
Strongly agree
Agree
Undecided
Disagree
Strongly disagree
If a patient presents to the ED repeatedly asking for more pain medication, this could be due to a missed diagnosis of the underlying pain source.
Strongly agree
Agree
Undecided
Disagree
Strongly disagree
I ask my patients about the severity of their pain.
Strongly agree
Agree
Undecided
Disagree
Strongly disagree
I include patient-reported pain levels in my notes.
Strongly agree
Agree
Undecided
Disagree
Strongly disagree
I think that proper pain management is associated with better patient outcomes.
Strongly agree
Agree
Undecided
Disagree
Strongly disagree
Case-based scenarios
For an adult presenting with chronic low back pain, I would initially prescribe: (circle only one)
NSAIDs
Tramadol
Duloxetine
Oxycodone
A 25-year-old female presents to the office with an acute episodic migraine According to the American Headache Society 2015 Guidelines, what treatment has Level A evidence?
Chlorpromazine IV 12.5 mg
Celecoxib 400 mg
Codeine/acetaminophen 25/400 mg
Naratriptan 2.5 mg
Codeine 30 mg
A 65-year-old man returns to the clinic for joint pain in his knees. He has a history of osteoarthritis and states that it is difficult for him to complete daily tasks. His pain was not treated by NSAIDs or weight loss. What should be the next line of treatment?
0-10 tablets of 5mg Tramadol
0-10 tablets of 5mg Oxycodone
Acetaminophen
Exercise
Continue NSAIDS and weight loss therapy
A 35-year-old male presents to the office with nephrolithiasis. His eGFR is >90ml/min and he has no history of GI bleed. How would you initially treat his pain?
No pain medication
NSAIDS
0-10 tablets of 5mg Oxycodone
For patients experiencing mild pain, I usually initially prescribe (circle one)
NSAIDs
Tylenol
Opioid
For patients experiencing moderate pain, I usually initially prescribe
(circle one)
NSAIDs
Tylenol
Opioid
For patients experiencing severe pain, I usually initially prescribe
(circle only one)
NSAID
Tylenol
Opioid

**Table 8:** EM resident pre-test data PGY: post-graduate year; DEA: Drug Enforcement Administration; PDMP: prescription drug monitoring program; PA: Pennsylvania; NSAID: non-steroidal anti-inflammatory drug; EM: emergency medicine

1. Please select your current level of training:	2. Do you hold a DEA License?	3. How satisfied are you with your current level of opioid-prescribing training?	4. When did you receive your opioid-prescribing training? (Select all that apply)	5. What are the three most common chief complaints for adults in the ED that were discharged with opioids? (select three)	6. Which three states have the highest percentage of opioid-related deaths per capita: (circle 3 states)	7. In 2017, how many drug overdose deaths were due to opioids?	8. In 2017, how many deaths were a result of heroin overdose?	9. Nearly half of all opioid related overdoses are due to valid prescription opioids.	10. What is the PDMP?	11. How often should the PDMP be referenced?	12. For an adult patient that presents to the emergency room with acute pain, according to current PA state guidelines, what is the maximum duration (days) for which an opioid prescription should be given?	13. When prescribing opioids to a minor, according to current PA state guidelines, the provider should:	14. For an adult presenting with noncancer pain, what should be the first course of action prior to formulating a pain control plan? (circle only one)	15. For an adult presenting to the ED with acute low back pain, I would typically prescribe: (circle only one)	16. A 25-year-old female presents to the office with an acute episodic migraine According to the American Headache Society 2015 Guidelines, what treatment has Level A evidence?	17. A 30-year-old male who actively uses IV heroin presents to the ED for a localized skin infection. After several hours, he begins to complain of anxiety and GI upset. You suspect opioid withdrawal and calculate his Clinical Opiate Withdrawal Score (COWS), which at 30 is rated “moderately severe”. How would you treat his current withdrawal symptoms?	18. For patients experiencing mild pain, I initially prescribe (circle one)	19. For patients experiencing moderate pain, I initially prescribe (circle one)	20. For patients experiencing severe pain, I initially prescribe (circle only one)	21. Opioids are effective in pain management.	22. Every patient that presents to the ED with pain should receive opioids.	23. I feel comfortable in my knowledge of non-opioid pain management.	24. If I suspect someone is abusing drugs, I will not prescribe them short-acting opioids.	25. Patient gender may affect my judgement of a patient's pain intensity	26. Patient race may affect my judgement of a patient's pain intensity	27. If a patient presents to the ED repeatedly asking for more pain medication, this could be due to a missed diagnosis of the underlying pain source.	28. I ask my patients about the severity of their pain.	29. I include patient-reported pain levels in my notes.
PGY2	No	Neutral	Medical School, Residency	Urolithiasis, Back pain	Kentucky, Pennsylvania, West Virginia	25,000	45,000	TRUE	Prescription Drug Monitoring Program	Anytime an opioid prescription is given.	7 days	All of the above	Consult the state monitoring program (PDMP)	Only non-opioid pain medications	Chlorpromazine IV 12.5 mg	Extended-release oxycodone	NSAIDs	NSAIDs	Opioid	Agree	Disagree	Agree	Agree	Agree	Agree	Undecided	Agree	Undecided
PGY2	No	Neutral	Never received any formal training	Headache, Abdominal pain, Back pain	California, New York, Pennsylvania	75,000	25,000	TRUE	Prescription Drug Monitoring Program	Anytime an opioid prescription is given.	7 days	All of the above	Short acting opioids	Only non-opioid pain medications	Chlorpromazine IV 12.5 mg	Buprenorphine-naloxone to bridge him to outpatient treatment	NSAIDs	NSAIDs	NSAID	Agree	Strongly disagree	Agree	Agree	Disagree	Disagree	Agree	Strongly agree	Strongly agree
PGY2	No	Very satisfied	Medical School, Residency, Personal reading	Headache, Abdominal pain, Back pain	New York, Pennsylvania, West Virginia	25,000	45,000	TRUE	Prescription Drug Monitoring Program	Anytime an opioid prescription is given.	3 days	All of the above	Consult the state monitoring program (PDMP)	Only non-opioid pain medications	Chlorpromazine IV 12.5 mg	Buprenorphine-naloxone to bridge him to outpatient treatment	NSAIDs	NSAIDs	NSAID	Disagree	Strongly disagree	Strongly agree	Agree	Agree	Agree	Agree	Strongly disagree	Strongly disagree
PGY1	No	Neutral	Residency	Dental pain, Urolithiasis	Alabama, Pennsylvania, West Virginia	45,000	45,000	FALSE	Prescription Drug Monitoring Program	Anytime an opioid prescription is given.	7 days	All of the above	Consult the state monitoring program (PDMP)	Only non-opioid pain medications	Chlorpromazine IV 12.5 mg	Buprenorphine-naloxone to bridge him to outpatient treatment	Tylenol	NSAIDs	Opioid	Agree	Disagree	Strongly disagree	Agree	Disagree	Disagree	Agree	Agree	Agree
PGY3	No	Unsatisfied	Residency, Personal reading	Dental pain, Urolithiasis, Back pain	Alabama, Kentucky, West Virginia	45,000	25,000	TRUE	Prescription Drug Monitoring Program	Anytime an opioid prescription is given.	7 days	All of the above	Only non-opioid pain medications	Only non-opioid pain medications	Celecoxib 400 mg	Buprenorphine-naloxone to bridge him to outpatient treatment	Tylenol	Tylenol	Opioid	Agree	Strongly disagree	Undecided	Disagree	Disagree	Disagree	Agree	Agree	Undecided
PGY1	No	Unsatisfied	Medical School, Residency	Dental pain, Urolithiasis, Back pain	Kentucky, South Carolina, West Virginia	75,000	45,000	TRUE	Prescription Drug Monitoring Program	Anytime an opioid prescription is given.	14 days	All of the above	Only non-opioid pain medications	Only non-opioid pain medications	Chlorpromazine IV 12.5 mg	Buprenorphine-naloxone to bridge him to outpatient treatment	Tylenol	Tylenol	Opioid	Agree	Undecided	Undecided	Disagree	Agree	Strongly agree	Agree	Agree	Agree
PGY3	No	Neutral	Medical School, Residency, Personal reading	Dental pain, Abdominal pain, Back pain	Alabama, Kentucky, West Virginia	75,000	45,000	TRUE	Prescription Drug Monitoring Program	Anytime an opioid prescription is given.	3 days	All of the above	Only non-opioid pain medications	Only non-opioid pain medications	Codeine/acetaminophen 25/400 mg	Buprenorphine-naloxone to bridge him to outpatient treatment	Tylenol	NSAIDs	Opioid	Agree	Disagree	Agree	Undecided	Agree	Agree	Agree	Agree	Undecided
PGY2	No	Satisfied	Medical School, Residency, Personal reading	Dental pain, Urolithiasis, Back pain	Kentucky, Pennsylvania, West Virginia	45,000	25,000	TRUE	Prescription Drug Monitoring Program	Anytime an opioid prescription is given.	3 days	All of the above	Consult the state monitoring program (PDMP)	Only non-opioid pain medications	Naratriptan 2.5 mg	Buprenorphine-naloxone to bridge him to outpatient treatment	NSAIDs	NSAIDs	Opioid	Agree	Strongly disagree	Agree	Agree	Disagree	Disagree	Agree	Agree	Agree
PGY3	No	Satisfied	Residency	Dental pain, Urolithiasis, Back pain	Alabama, Kentucky, West Virginia	25,000	45,000	TRUE	Prescription Drug Monitoring Program	Anytime an opioid prescription is given.	3 days	All of the above	Only non-opioid pain medications	Only non-opioid pain medications	Naratriptan 2.5 mg	Buprenorphine-naloxone to bridge him to outpatient treatment	Tylenol	NSAIDs	NSAID	Undecided	Strongly disagree	Agree	Agree	Disagree	Disagree	Agree	Disagree	Undecided
PGY3	No	Satisfied	Personal reading	Dental pain, Urolithiasis, Back pain	Delaware, Pennsylvania, West Virginia	75,000	25,000	TRUE	Prescription Drug Monitoring Program	Anytime an opioid prescription is given.	3 days	All of the above	Consult the state monitoring program (PDMP)	Only non-opioid pain medications	Chlorpromazine IV 12.5 mg	Buprenorphine-naloxone to bridge him to outpatient treatment	NSAIDs	NSAIDs	Opioid	Agree	Disagree	Agree	Strongly agree	Strongly disagree	Strongly disagree	Agree	Strongly agree	Agree
PGY2	No	Very satisfied	Medical School, Residency	Dental pain, Urolithiasis, Back pain	Kentucky, Pennsylvania, West Virginia	45,000	45,000	TRUE	Prescription Drug Monitoring Program	Anytime an opioid prescription is given.	3 days	All of the above	Consult the state monitoring program (PDMP)	Only non-opioid pain medications	Naratriptan 2.5 mg	Oral morphine	NSAIDs	NSAIDs	NSAID	Strongly agree	Strongly disagree	Agree	Strongly disagree	Strongly agree	Strongly agree	Strongly agree	Strongly disagree	Strongly disagree
PGY2	No	Unsatisfied	Medical School, Residency	Back pain	California, New York, Pennsylvania	25,000	45,000	TRUE	Prescription Drug Monitoring Program	Anytime an opioid prescription is given.	3 days	All of the above	Only non-opioid pain medications	Only non-opioid pain medications	Celecoxib 400 mg	Buprenorphine-naloxone to bridge him to outpatient treatment	Tylenol	NSAIDs	NSAID	Strongly agree	Disagree	Strongly agree	Strongly agree	Strongly disagree	Strongly disagree	Agree	Strongly agree	Agree
PGY3	No	Satisfied	Medical School, Residency	Urolithiasis	Kentucky, Pennsylvania, West Virginia	45,000	45,000	TRUE	Prescription Drug Monitoring Program	Anytime an opioid prescription is given.	3 days	All of the above	Only non-opioid pain medications	Only non-opioid pain medications	Chlorpromazine IV 12.5 mg	Buprenorphine-naloxone to bridge him to outpatient treatment	NSAIDs	NSAIDs	Opioid	Agree	Strongly disagree	Agree	Agree	Strongly disagree	Strongly disagree	Agree	Strongly agree	Agree
PGY3	No	Neutral	Medical School, Residency	Dental pain, Urolithiasis, Back pain	Alabama, Pennsylvania, South Carolina	45,000	15,000	TRUE	Prescription Drug Monitoring Program	Anytime an opioid prescription is given.	No limit	All of the above	Consult the state monitoring program (PDMP)	Only non-opioid pain medications	Chlorpromazine IV 12.5 mg	Buprenorphine-naloxone to bridge him to outpatient treatment	Tylenol	NSAIDs	NSAID	Disagree	Strongly disagree	Undecided	Strongly agree	Strongly disagree	Strongly disagree	Agree	Agree	Undecided
PGY1	No	Satisfied	Medical School, Residency, Personal reading	Dental pain, Urolithiasis, Back pain	Kentucky, Pennsylvania, West Virginia	75,000	45,000	TRUE	Prescription Drug Monitoring Program	Anytime an opioid prescription is given.	3 days	All of the above	Only non-opioid pain medications	Only non-opioid pain medications	Naratriptan 2.5 mg	Buprenorphine-naloxone to bridge him to outpatient treatment	Tylenol	NSAIDs	Opioid	Strongly agree	Strongly disagree	Agree	Undecided	Strongly disagree	Strongly disagree	Agree	Strongly agree	Strongly agree
PGY1	No	Very unsatisfied	Personal reading	Dental pain, Abdominal pain, Urolithiasis	California, Pennsylvania, West Virginia	45,000	75,000	FALSE	Prescription Drug Monitoring Program	Anytime an opioid prescription is given.	3 days	All of the above	Extended-released schedule II products	Only non-opioid pain medications	Chlorpromazine IV 12.5 mg	Oral morphine	Tylenol	Tylenol	Tylenol	Agree	Strongly disagree	Disagree	Disagree	Agree	Agree	Undecided	Strongly agree	Agree
PGY2	No	Neutral	Residency	Dental pain, Urolithiasis, Back pain	Kentucky, Pennsylvania, West Virginia	25,000	25,000	TRUE	Prescription Drug Monitoring Program	Anytime an opioid prescription is given.	1 day	All of the above	Only non-opioid pain medications	Only non-opioid pain medications	Celecoxib 400 mg	Buprenorphine-naloxone to bridge him to outpatient treatment	NSAIDs	NSAIDs	NSAID	Agree	Disagree	Undecided	Strongly agree	Undecided	Undecided	Agree	Agree	Agree
PGY1	No	Neutral	Medical School	Dental pain, Abdominal pain, Back pain	Alabama, Pennsylvania, West Virginia	45,000	25,000	FALSE	Prescription Drug Monitoring Program	Anytime an opioid prescription is given.	7 days	All of the above	Only non-opioid pain medications	Only non-opioid pain medications	Chlorpromazine IV 12.5 mg	Buprenorphine-naloxone to bridge him to outpatient treatment	Tylenol	NSAIDs	Opioid	Agree	Strongly disagree	Agree	Agree	Strongly disagree	Strongly disagree	Agree	Agree	Agree
PGY1	No	Neutral	Residency	Back pain	New York, Delaware, Pennsylvania	75,000	45,000	TRUE	Prescription Drug Monitoring Program	Anytime an opioid prescription is given.	3 days	All of the above	Only non-opioid pain medications	Only non-opioid pain medications	Celecoxib 400 mg	Buprenorphine-naloxone to bridge him to outpatient treatment	Tylenol	Tylenol	Tylenol	Strongly agree	Strongly disagree	Strongly agree	Strongly agree	Strongly disagree	Strongly disagree	Disagree	Strongly agree	Strongly agree
PGY1	No	Satisfied	Medical School, Residency, Personal reading	Dental pain, Abdominal pain, Urolithiasis	Delaware, Pennsylvania, West Virginia	75,000	75,000	TRUE	Prescription Drug Monitoring Program	Anytime an opioid prescription is given.	3 days	All of the above	Only non-opioid pain medications	Only non-opioid pain medications	Celecoxib 400 mg	Buprenorphine-naloxone to bridge him to outpatient treatment	Tylenol	NSAIDs	Opioid	Agree	Strongly disagree	Agree	Agree	Disagree	Disagree	Agree	Agree	Agree
PGY3	No	Neutral	Residency	Dental pain, Urolithiasis, Back pain	Kentucky, Pennsylvania, West Virginia	25,000	45,000	TRUE	Prescription Drug Monitoring Program	Anytime an opioid prescription is given.	3 days	All of the above	Only non-opioid pain medications	31-40 tablets of 5mg Oxycodone + NSAID	Chlorpromazine IV 12.5 mg	Buprenorphine-naloxone to bridge him to outpatient treatment	NSAIDs	NSAIDs	Opioid	Agree	Disagree	Undecided	Disagree	Strongly disagree	Strongly disagree	Agree	Agree	Undecided
PGY1	No	Neutral	Never received any formal training	Abdominal pain, Urolithiasis, Back pain	Alabama, Kentucky, West Virginia	75,000	45,000	TRUE	Prescription Drug Monitoring Program	Anytime an opioid prescription is given.	7 days	All of the above	Only non-opioid pain medications	Only non-opioid pain medications	Chlorpromazine IV 12.5 mg	Buprenorphine-naloxone to bridge him to outpatient treatment	NSAIDs	NSAIDs	Opioid	Agree	Strongly disagree	Agree	Agree	Strongly disagree	Strongly disagree	Agree	Strongly agree	Strongly agree
PGY3	No	Satisfied	Medical School, Residency, Personal reading	Abdominal pain, Urolithiasis, Back pain	Delaware, Pennsylvania, West Virginia	75,000	45,000	TRUE	Prescription Drug Monitoring Program	Anytime an opioid prescription is given.	3 days	All of the above	Consult the state monitoring program (PDMP)	Only non-opioid pain medications	Celecoxib 400 mg	Buprenorphine-naloxone to bridge him to outpatient treatment	Tylenol	NSAIDs	Opioid	Agree	Strongly disagree	Agree	Agree	Disagree	Disagree	Agree	Agree	Agree
PGY3	No	Very satisfied	Residency	Dental pain, Abdominal pain, Back pain	California, Pennsylvania, South Carolina	75,000	25,000	TRUE	Prescription Drug Monitoring Program	Anytime an opioid prescription is given.	3 days	All of the above	Only non-opioid pain medications	Only non-opioid pain medications	Chlorpromazine IV 12.5 mg	Buprenorphine-naloxone to bridge him to outpatient treatment	Tylenol	NSAIDs	Opioid	Agree	Disagree	Agree	Agree	Strongly disagree	Strongly disagree	Agree	Undecided	Agree
PGY2	No	Neutral	Medical School, Residency	Dental pain, Urolithiasis, Back pain	Delaware, Pennsylvania, West Virginia	75,000	45,000	TRUE	Prescription Drug Monitoring Program	Anytime an opioid prescription is given.	No limit	All of the above	Consult the state monitoring program (PDMP)	Only non-opioid pain medications	Chlorpromazine IV 12.5 mg	Buprenorphine-naloxone to bridge him to outpatient treatment	NSAIDs	NSAIDs	Opioid	Agree	Strongly disagree	Agree	Strongly agree	Agree	Agree	Strongly agree	Strongly agree	Agree
PGY3	No	Satisfied	Medical School, Residency, Personal reading	Dental pain, Urolithiasis, Back pain	Kentucky, Pennsylvania, West Virginia	45,000	25,000	TRUE	Prescription Drug Monitoring Program	Anytime an opioid prescription is given.	3 days	All of the above	Consult the state monitoring program (PDMP)	Only non-opioid pain medications	Chlorpromazine IV 12.5 mg	Buprenorphine-naloxone to bridge him to outpatient treatment	Tylenol	NSAIDs	Opioid	Strongly agree	Strongly disagree	Strongly agree	Strongly agree	Agree	Agree	Agree	Agree	Agree
PGY3	No	Very satisfied	Medical School, Residency	Urolithiasis			75,000	TRUE	Prescription Drug Monitoring Program	Anytime an opioid prescription is given.	3 days	All of the above	Consult the state monitoring program (PDMP)	Only non-opioid pain medications	Chlorpromazine IV 12.5 mg	Buprenorphine-naloxone to bridge him to outpatient treatment	Tylenol	Tylenol	Tylenol	Strongly agree	Strongly disagree	Strongly agree	Strongly disagree	Strongly disagree	Strongly disagree	Agree	Agree	Agree

**Table 9:** EM resident post-test data PGY: post-graduate year; DEA: Drug Enforcement Administration; PDMP: prescription drug monitoring program; PA: Pennsylvania; NSAID: non-steroidal anti-inflammatory drug; EM: emergency medicine

1. Please select your current level of training:	2. Do you hold a DEA License?	3. How satisfied are you with your current level of opioid-prescribing training?	4. When did you receive your opioid-prescribing training? (Select all that apply)	5. What are the three most common chief complaints for adults in the ED that were discharged with opioids? (select three)	6. Which three states have the highest percentage of opioid-related deaths per capita: (circle 3 states)	7. In 2017, how many drug overdose deaths were due to opioids?	8. In 2017, how many deaths were a result of heroin overdose?	9. Nearly half of all opioid related overdoses are due to valid prescription opioids.	10. What is the PDMP?	11. How often should the PDMP be referenced?	12. For an adult patient that presents to the emergency room with acute pain, according to current PA state guidelines, what is the maximum duration (days) for which an opioid prescription should be given?	13. When prescribing opioids to a minor, according to current PA state guidelines, the provider should:	14. For an adult presenting with noncancer pain, what should be the first course of action prior to formulating a pain control plan? (circle only one)	15. For an adult presenting to the ED with acute low back pain, I would typically prescribe: (circle only one)	16. A 25-year-old female presents to the office with an acute episodic migraine According to the American Headache Society 2015 Guidelines, what treatment has Level A evidence?	17. A 30-year-old male who actively uses IV heroin presents to the ED for a localized skin infection. After several hours, he begins to complain of anxiety and GI upset. You suspect opioid withdrawal and calculate his Clinical Opiate Withdrawal Score (COWS), which at 30 is rated “moderately severe”. How would you treat his current withdrawal symptoms?	18. For patients experiencing mild pain, I initially prescribe (circle one)	19. For patients experiencing moderate pain, I initially prescribe (circle one)	20. For patients experiencing severe pain, I initially prescribe (circle only one)	21. Opioids are effective in pain management.	22. Every patient that presents to the ED with pain should receive opioids.	23. I feel comfortable in my knowledge of non-opioid pain management.	24. If I suspect someone is abusing drugs, I will not prescribe them short-acting opioids.	25. Patient gender may affect my judgement of a patient's pain intensity	26. Patient race may affect my judgement of a patient's pain intensity	27. If a patient presents to the ED repeatedly asking for more pain medication, this could be due to a missed diagnosis of the underlying pain source.	28. I ask my patients about the severity of their pain.	29. I include patient-reported pain levels in my notes.
PGY2	No	Satisfied	Medical School, Residency	Dental pain, Urolithiasis, Back pain	Delaware, Pennsylvania, West Virginia	75,000	45,000	TRUE	Prescription Drug Monitoring Program	Anytime an opioid prescription is given.	7 days	All of the above	Consult the state monitoring program (PDMP)	Only non-opioid pain medications	Chlorpromazine IV 12.5 mg	NSAIDs	NSAIDs	NSAIDs	Opioid	Agree	Strongly disagree	Agree	Strongly agree	Agree	Agree	Strongly agree	Agree	Agree
PGY3	No	Very satisfied	Residency	Urolithiasis	Kentucky, Pennsylvania, West Virginia	75,000	25,000	TRUE	Prescription Drug Monitoring Program	Anytime an opioid prescription is given.	3 days	All of the above	Only non-opioid pain medications	Only non-opioid pain medications	Chlorpromazine IV 12.5 mg	Buprenorphine-naloxone to bridge him to outpatient treatment	Tylenol	Tylenol	Tylenol	Strongly agree	Strongly disagree	Strongly agree	Disagree	Strongly disagree	Strongly disagree	Agree	Agree	Agree
PGY3	No	Very satisfied	Residency	Urolithiasis	Kentucky, Pennsylvania, West Virginia	75,000	25,000	TRUE	Prescription Drug Monitoring Program	Anytime an opioid prescription is given.	3 days	All of the above	Only non-opioid pain medications	Only non-opioid pain medications	Chlorpromazine IV 12.5 mg	Buprenorphine-naloxone to bridge him to outpatient treatment	Tylenol	Tylenol	Tylenol	Strongly agree	Strongly disagree	Strongly agree	Disagree	Strongly disagree	Strongly disagree	Agree	Agree	Agree
PGY1	No	Satisfied	Medical School, Residency, Personal reading	Dental pain, Urolithiasis, Back pain	Kentucky, Delaware, Pennsylvania, West Virginia	75,000	15,000	TRUE	Prescription Drug Monitoring Program	Anytime an opioid prescription is given.	3 days	All of the above	Only non-opioid pain medications	Only non-opioid pain medications	Naratriptan 2.5 mg	Buprenorphine-naloxone to bridge him to outpatient treatment	Tylenol	NSAIDs	Opioid	Agree	Strongly disagree	Agree	Undecided	Strongly disagree	Strongly disagree	Strongly agree	Strongly agree	Strongly agree
PGY3	No	Satisfied	Medical School, Residency, Personal reading	Dental pain, Abdominal pain, Urolithiasis	Delaware, Pennsylvania, West Virginia	75,000	45,000	TRUE	Prescription Drug Monitoring Program	Anytime an opioid prescription is given.	7 days	All of the above	Consult the state monitoring program (PDMP)	0-10 tablets of 5mg Oxycodone + NSAID	Naratriptan 2.5 mg	Buprenorphine-naloxone to bridge him to outpatient treatment	Tylenol	NSAIDs	Opioid	Agree	Strongly disagree	Agree	Disagree	Disagree	Disagree	Agree	Agree	Agree
PGY2	No	Satisfied	Medical School, Residency	Abdominal pain, Urolithiasis, Back pain	California, New York, Pennsylvania	75,000	15,000	TRUE	Prescription Drug Monitoring Program	Anytime an opioid prescription is given.	7 days	All of the above	Consult the state monitoring program (PDMP)	Only non-opioid pain medications	Naratriptan 2.5 mg	Buprenorphine-naloxone to bridge him to outpatient treatment	Tylenol	NSAIDs	Opioid	Strongly agree	Strongly disagree	Agree	Strongly agree	Disagree	Disagree	Agree	Strongly agree	Strongly agree
PGY1	No	Neutral	Medical School, Residency, Personal reading	Abdominal pain	California, South Carolina, West Virginia	25,000	15,000	TRUE	Prescription Drug Monitoring Program	Anytime an opioid prescription is given.	3 days	All of the above	Only non-opioid pain medications	Only non-opioid pain medications	Naratriptan 2.5 mg	Buprenorphine-naloxone to bridge him to outpatient treatment	Tylenol	NSAIDs	Opioid	Strongly agree	Disagree	Agree	Disagree	Disagree	Disagree	Agree	Agree	Agree
PGY1	No	Neutral	Residency	Dental pain, Urolithiasis, Back pain	New York, Delaware, Pennsylvania	45,000	15,000	TRUE	Prescription Drug Monitoring Program	Anytime an opioid prescription is given.	3 days	All of the above	Consult the state monitoring program (PDMP)	Only non-opioid pain medications	Chlorpromazine IV 12.5 mg	Buprenorphine-naloxone to bridge him to outpatient treatment	Tylenol	Tylenol	NSAID	Strongly agree	Strongly disagree	Undecided	Undecided	Undecided	Undecided	Agree	Agree	Agree
PGY3	No	Satisfied	Residency	Dental pain, Urolithiasis, Back pain	California, New York, Pennsylvania	45,000	25,000	TRUE	Prescription Drug Monitoring Program	Anytime an opioid prescription is given.	3 days	All of the above	Only non-opioid pain medications	Only non-opioid pain medications	Celecoxib 400 mg	Buprenorphine-naloxone to bridge him to outpatient treatment	Tylenol	NSAIDs	Opioid	Agree	Strongly disagree	Agree	Agree	Strongly agree	Strongly agree	Agree	Disagree	Disagree
PGY1		Neutral	College	Abdominal pain	California, Kentucky, New York	15,000	25,000	FALSE	Prescribing Directory of Medical Providers	Anytime an opioid prescription is given.	3 days	All of the above	Short acting opioids	0-10 tablets of 5mg Oxycodone + NSAID	Codeine/acetaminophen 25/400 mg	Tylenol	Tylenol	Tylenol	Tylenol	Agree	Agree	Undecided	Undecided	Undecided	Undecided	Undecided	Undecided	Undecided
PGY1	No	Neutral	Never received any formal training	Dental pain, Abdominal pain, Urolithiasis	Kentucky, Pennsylvania, West Virginia	75,000	45,000	TRUE	Prescription Drug Monitoring Program	Anytime an opioid prescription is given.	3 days	All of the above	Only non-opioid pain medications	Only non-opioid pain medications	Chlorpromazine IV 12.5 mg	Tylenol	NSAIDs	NSAIDs	Opioid	Strongly agree	Disagree	Disagree	Agree	Disagree	Disagree	Agree	Agree	Agree
PGY3	No	Satisfied	Medical School, Residency	Chest pain, Abdominal pain, Back pain	Kentucky, Delaware, Pennsylvania	25,000	25,000	FALSE	Prescription Drug Monitoring Program	Anytime an opioid prescription is given.	3 days	All of the above	Only non-opioid pain medications	Only non-opioid pain medications	Chlorpromazine IV 12.5 mg	Buprenorphine-naloxone to bridge him to outpatient treatment	NSAIDs	NSAIDs	Opioid	Agree	Strongly disagree	Strongly agree	Undecided	Strongly disagree	Strongly disagree	Agree	Strongly agree	Agree
PGY3	No	Neutral	Residency	Dental pain, Urolithiasis, Back pain	Kentucky, Pennsylvania, West Virginia	45,000	25,000	TRUE	Prescription Drug Monitoring Program	Anytime an opioid prescription is given.	7 days	All of the above	Only non-opioid pain medications	Only non-opioid pain medications	Naratriptan 2.5 mg	Buprenorphine-naloxone to bridge him to outpatient treatment	NSAIDs	Tylenol	Opioid	Strongly agree	Strongly disagree	Agree	Disagree	Agree	Agree	Agree	Strongly agree	Agree

**Table 10:** IM resident pre-test data PGY: post-graduate year; DEA: Drug Enforcement Administration; PDMP: prescription drug monitoring program; PA: Pennsylvania; NSAID: non-steroidal anti-inflammatory drug; IM: internal medicine

1. Please select your current level of training:	2. Do you hold a DEA License?	3. How satisfied are you with your current level of opioid-prescribing training?	4. When did you receive your opioid-prescribing training? (Select all that apply)	5. Which three states have the highest percentage of opioid-related deaths per capita:(circle 3 states)	6. In 2017, how many drug overdose deaths were due to opioids?	7. In 2017, how many deaths were a result of heroin overdose?	8. Nearly half of all opioid related overdoses are due to valid prescription opioids.	9. What is the PDMP?	10. How often should the PDMP be referenced?	11. Opioids are effective in pain management.	12. Every patient that presents to the office with pain should receive opioids.	13. I feel comfortable in my knowledge of non-opioid pain management.	14. If I suspect someone is abusing opioids, I do not prescribe opioids to them.	15. Patient gender may affect my judgement of a patient's pain intensity	16. Patient race may affect my judgement of a patient's pain intensity	17. If a patient presents to the ED repeatedly asking for more pain medication, this could be due to a missed diagnosis of the underlying pain source.	17. If a patient presents to the ED repeatedly asking for more pain medication, this could be due to a missed diagnosis of the underlying pain source.	18. I ask my patients about the severity of their pain.	19. I include patient-reported pain levels in my notes.	20. I think that proper pain management is associated with better patient outcomes.	21. For an adult presenting with chronic low back pain, I would initially prescribe: (circle only one)	22. A 25-year-old female presents to the office with an acute episodic migraine According to the American Headache Society 2015 Guidelines, what treatment has Level A evidence?	23. A 65-year-old man returns to the clinic for joint pain in his knees. He has a history of osteoarthritis and states that it is difficult for him to complete daily tasks. His pain was not treated by NSAIDs or weight loss. What should be the next line of treatment?	24. A 35-year-old male presents to the office with nephrolithiasis. His eGFR is >90ml/min and he has no history of GI bleed. How would you initially treat his pain?	25. For patients experiencing mild pain, I usually initially prescribe (circle one)	26. For patients experiencing moderate pain, I usually initially prescribe (circle one)	27. For patients experiencing severe pain, I usually initially prescribe (circle only one)
PGY3	No	Unsatisfied	Never received any formal training	Alabama, Kentucky, West Virginia	75,000	45,000	TRUE	Prescription Drug Monitoring Program	Anytime an opioid prescription is given.	Strongly agree	Strongly disagree	Agree	Agree	Disagree	Disagree		Agree	Agree	Agree	Agree	NSAIDs	Celecoxib 400 mg	Continue NSAIDS and weight loss therapy	NSAIDS	Tylenol	Tylenol	Opioid
PGY2	No	Neutral	Never received any formal training	California, New York, Pennsylvania	75,000	45,000	TRUE	Prescription Drug Monitoring Program	Anytime an opioid prescription is given.	Undecided	Strongly disagree	Disagree	Agree	Disagree	Disagree		Agree	Agree	Agree	Agree	NSAIDs	Naratriptan 2.5 mg	0-10 tablets of 5mg Tramadol	0-10 tablets of 5mg Oxycodone	Tylenol	Tylenol	Opioid
PGY3	No	Very satisfied	Medical School, Residency, Personal reading	Alabama, Pennsylvania, West Virginia	75,000	45,000	TRUE	Prescription Drug Monitoring Program	Anytime an opioid prescription is given.	Undecided	Strongly disagree	Strongly agree	Agree	Disagree	Strongly disagree		Strongly agree	Strongly agree	Strongly agree	Strongly agree	NSAIDs	Chlorpromazine IV 12.5 mg	Continue NSAIDS and weight loss therapy	NSAIDS	Tylenol	NSAIDs	NSAIDs
PGY1	No	Unsatisfied	Medical School, Residency	Kentucky, New York, Pennsylvania	45,000	45,000	TRUE	Prescription Drug Monitoring Program	Anytime an opioid prescription is given.	Strongly agree	Strongly disagree	Disagree	Agree	Agree	Agree		Agree	Strongly agree	Strongly agree	Strongly agree	NSAIDs	Celecoxib 400 mg	Acetaminophen	NSAIDS	Tylenol	Tylenol	Opioid
PGY3	No	Neutral	Residency	California, Pennsylvania, West Virginia	45,000	25,000	TRUE	Prescription Drug Monitoring Program	Anytime an opioid prescription is given.	Agree	Disagree	Agree	Strongly agree	Disagree	Strongly disagree		Agree	Agree	Undecided	Agree	NSAIDs	Naratriptan 2.5 mg	Acetaminophen	NSAIDS	Tylenol	Tylenol	Opioid
PGY3	No	Very satisfied	Medical School, Residency, Personal reading	Kentucky, South Carolina, West Virginia	45,000	45,000	TRUE	Prescribing Directory of Medical Providers	Anytime an opioid prescription is given.	Disagree	Strongly disagree	Strongly agree	Agree	Agree	Agree		Agree	Agree	Disagree	Strongly agree	NSAIDs	Celecoxib 400 mg	Continue NSAIDS and weight loss therapy	NSAIDS	Tylenol	NSAIDs	Opioid
PGY3	No	Satisfied	Residency	New York, Delaware, Pennsylvania	75,000	45,000	TRUE	Prescription Drug Monitoring Program	Anytime an opioid prescription is given.	Strongly agree	Strongly disagree	Agree	Agree	Disagree	Disagree		Agree	Agree	Disagree	Agree	NSAIDs	Celecoxib 400 mg	Acetaminophen	NSAIDS	Tylenol	Tylenol	Tylenol
PGY3	No	Unsatisfied	Medical School, Residency, Personal reading	Pennsylvania, South Carolina, West Virginia	45,000	15,000	TRUE	Prescription Drug Monitoring Program	Anytime an opioid prescription is given.	Agree	Strongly disagree	Agree	Agree	Agree	Undecided		Agree	Agree	Agree	Strongly agree	NSAIDs	Naratriptan 2.5 mg	Exercise	NSAIDS	Tylenol	NSAIDs	Opioid
PGY2	No	Neutral	Medical School, Residency	Kentucky, Pennsylvania, West Virginia	75,000	45,000	TRUE	Prescription Drug Monitoring Program	Anytime an opioid prescription is given.	Strongly agree	Strongly disagree	Agree	Agree	Strongly disagree	Strongly disagree		Agree	Agree	Agree	Agree	NSAIDs	Naratriptan 2.5 mg	Acetaminophen	NSAIDS	NSAIDs	NSAIDs	Opioid
PGY2	No	Very unsatisfied	Medical School	California, New York, Pennsylvania	75,000	25,000	TRUE	Prescription Drug Monitoring Program	Anytime an opioid prescription is given.	Strongly disagree	Strongly disagree	Agree	Strongly disagree	Strongly disagree	Strongly disagree		Strongly agree	Strongly agree	Strongly agree	Strongly agree	NSAIDs	Chlorpromazine IV 12.5 mg	Acetaminophen	NSAIDS	NSAIDs	NSAIDs	NSAIDs
PGY2	No	Unsatisfied	Residency	California, Pennsylvania, West Virginia	25,000	45,000	TRUE	Prescription Drug Monitoring Program	Anytime an opioid prescription is given.	Agree	Strongly disagree	Agree	Agree	Agree	Agree		Agree	Agree	Agree	Strongly agree	NSAIDs	Naratriptan 2.5 mg	Exercise	NSAIDS	NSAIDs	NSAIDs	NSAIDs
PGY3	Yes	Neutral	Residency	New York, Pennsylvania, West Virginia	75,000	75,000	TRUE	Prescription Drug Monitoring Program	Anytime an opioid prescription is given.	Undecided	Disagree	Agree	Agree	Agree	Undecided		Agree	Agree	Agree	Agree	NSAIDs	Naratriptan 2.5 mg	Continue NSAIDS and weight loss therapy	0-10 tablets of 5mg Oxycodone	Tylenol	NSAIDs	Opioid
PGY1	No	Unsatisfied	Medical School	Kentucky, Pennsylvania, West Virginia	25,000	15,000	TRUE	Prescription Drug Monitoring Program	Anytime an opioid prescription is given.	Agree	Strongly disagree	Agree	Agree	Agree	Agree		Agree	Agree	Agree	Agree	NSAIDs	Celecoxib 400 mg	Continue NSAIDS and weight loss therapy	NSAIDS	Tylenol	NSAIDs	Opioid
PGY3	No	Satisfied	Medical School, Residency, Personal reading	Delaware, Pennsylvania, West Virginia	75,000	45,000	FALSE	Prescription Drug Monitoring Program	Anytime an opioid prescription is given.	Agree	Strongly disagree	Agree	Strongly agree	Agree	Agree		Agree	Strongly agree	Strongly agree	Strongly agree	NSAIDs	Chlorpromazine IV 12.5 mg	0-10 tablets of 5mg Tramadol	NSAIDS	Tylenol	NSAIDs	Tylenol
PGY1	No	Neutral	Medical School	California, Kentucky, West Virginia	75,000	45,000	TRUE	Prescription Drug Monitoring Program	Anytime an opioid prescription is given.	Agree	Strongly disagree	Agree	Disagree	Disagree	Strongly disagree		Strongly agree	Strongly agree	Strongly agree	Strongly agree	NSAIDs	Naratriptan 2.5 mg	Continue NSAIDS and weight loss therapy	0-10 tablets of 5mg Oxycodone	Tylenol	Tylenol	Tylenol
PGY2	No	Unsatisfied	Residency	California, New York, Pennsylvania	75,000	75,000	TRUE	Prescription Drug Monitoring Program	Anytime an opioid prescription is given.	Agree	Strongly disagree	Agree	Strongly agree	Agree	Agree		Undecided	Agree	Agree	Agree	NSAIDs	Celecoxib 400 mg	0-10 tablets of 5mg Tramadol	NSAIDS	Tylenol	NSAIDs	Opioid
PGY3	No	Unsatisfied	Medical School, Residency, Personal reading	Delaware, South Carolina, West Virginia	45,000	15,000	TRUE	Prescription Drug Monitoring Program	Anytime an opioid prescription is given.	Agree	Strongly disagree	Undecided	Agree	Disagree	Disagree		Agree	Agree	Undecided	Agree	NSAIDs	Celecoxib 400 mg	Exercise	0-10 tablets of 5mg Oxycodone	Tylenol	NSAIDs	Opioid
PGY3	No	Neutral	Medical School, Residency	California, New York, Pennsylvania	75,000	45,000	TRUE	Prescription Drug Monitoring Program	Anytime an opioid prescription is given.	Agree	Strongly disagree	Agree	Undecided	Strongly disagree	Undecided		Agree	Strongly agree	Strongly agree	Strongly agree	NSAIDs	Chlorpromazine IV 12.5 mg	Continue NSAIDS and weight loss therapy	NSAIDS	Tylenol	Tylenol	Opioid
PGY2	No	Neutral	Medical School, Residency, Personal reading	Alabama, Pennsylvania, West Virginia	45,000	15,000	TRUE	Prescription Drug Monitoring Program	Anytime an opioid prescription is given.	Agree	Strongly disagree	Disagree	Strongly agree	Undecided	Undecided		Agree	Agree	Agree	Agree		Chlorpromazine IV 12.5 mg	Acetaminophen		Tylenol	Tylenol	Opioid
PGY1	Yes	Satisfied	Medical School, Residency	New York, Delaware, Pennsylvania	45,000	25,000	TRUE	Prescription Drug Monitoring Program	Anytime an opioid prescription is given.	Agree	Strongly disagree	Undecided	Disagree	Undecided	Undecided		Agree	Strongly agree	Agree	Agree	NSAIDs	Celecoxib 400 mg	0-10 tablets of 5mg Tramadol	NSAIDS	Tylenol	Tylenol	Tylenol
PGY2	No	Unsatisfied	Never received any formal training	Alabama, Kentucky, South Carolina	45,000	25,000	TRUE	Prescription Drug Monitoring Program	Anytime an opioid prescription is given.	Agree	Disagree	Agree	Agree	Agree	Disagree		Agree	Agree	Agree	Agree	NSAIDs	Naratriptan 2.5 mg	0-10 tablets of 5mg Oxycodone	0-10 tablets of 5mg Oxycodone	Tylenol	Tylenol	Opioid
PGY3	No	Neutral	Medical School, Residency	Alabama, New York, Pennsylvania	45,000	25,000	TRUE	Prescription Drug Monitoring Program	Anytime an opioid prescription is given.	Agree	Strongly disagree	Agree	Agree	Strongly disagree	Strongly disagree		Agree	Disagree	Disagree	Agree	NSAIDs	Naratriptan 2.5 mg	Exercise	NSAIDS	Tylenol	Opioid	Opioid
PGY1	No	Neutral	Medical School, Residency	Alabama, Pennsylvania, West Virginia	75,000	45,000	TRUE	Prescription Drug Monitoring Program	Anytime an opioid prescription is given.	Undecided	Strongly disagree	Disagree	Undecided	Strongly disagree	Undecided		Agree	Agree	Agree	Strongly agree	NSAIDs	Celecoxib 400 mg	0-10 tablets of 5mg Tramadol	NSAIDS	Tylenol	Tylenol	Opioid
PGY1	No	Neutral	Medical School	Kentucky, Pennsylvania, West Virginia	45,000	45,000	TRUE	Prescription Drug Monitoring Program	Anytime an opioid prescription is given.	Agree	Strongly disagree	Agree	Undecided	Agree	Undecided		Agree	Agree	Agree	Strongly agree	NSAIDs	Naratriptan 2.5 mg	0-10 tablets of 5mg Tramadol	No pain medication	Tylenol	NSAIDs	NSAIDs
PGY1	No	Unsatisfied	Medical School	Kentucky, Pennsylvania, West Virginia	45,000	25,000	TRUE	Prescription Drug Monitoring Program	Anytime an opioid prescription is given.	Disagree	Strongly disagree	Undecided	Agree	Agree	Agree		Strongly agree	Agree	Disagree	Agree	NSAIDs	Naratriptan 2.5 mg	Acetaminophen	0-10 tablets of 5mg Oxycodone	Tylenol	NSAIDs	Opioid
PGY3	No	Neutral	Residency	California, New York, Pennsylvania	75,000	25,000	TRUE	Prescription Drug Monitoring Program	Anytime an opioid prescription is given.	Agree	Disagree	Agree	Agree	Disagree	Undecided		Undecided	Agree	Disagree	Agree	Duloxetine	Celecoxib 400 mg	Acetaminophen	NSAIDS	Tylenol	Tylenol	Tylenol
PGY1	No	Unsatisfied	Residency	Kentucky, Pennsylvania, West Virginia	45,000	15,000	TRUE	Prescription Drug Monitoring Program	Anytime an opioid prescription is given.	Undecided	Strongly disagree	Disagree	Agree	Strongly agree	Strongly agree		Agree	Strongly agree	Agree	Strongly agree	NSAIDs	Chlorpromazine IV 12.5 mg	Continue NSAIDS and weight loss therapy	NSAIDS	Tylenol	NSAIDs	Opioid
PGY1	No	Neutral	Medical School	New York, Delaware, West Virginia	75,000	75,000	FALSE	Prescription Drug Monitoring Program	Anytime an opioid prescription is given.	Agree	Strongly disagree	Undecided	Agree	Strongly disagree	Strongly disagree		Agree	Strongly agree	Agree	Agree	NSAIDs	Naratriptan 2.5 mg	Acetaminophen	0-10 tablets of 5mg Oxycodone	Tylenol	NSAIDs	Tylenol
PGY1	No	Unsatisfied	Residency	Kentucky, Pennsylvania, West Virginia	15,000	25,000	FALSE	Prescription Drug Monitoring Program	Anytime an opioid prescription is given.	Strongly agree	Strongly disagree	Disagree	Strongly agree	Strongly agree	Agree		Agree	Strongly agree	Strongly agree	Strongly agree	NSAIDs	Celecoxib 400 mg	0-10 tablets of 5mg Tramadol	NSAIDS	Tylenol	NSAIDs	Opioid
PGY3	No	Neutral	Residency	New York, Delaware, Pennsylvania	25,000	15,000	FALSE	Prescription Drug Monitoring Program	Anytime an opioid prescription is given.	Agree	Strongly disagree	Agree	Agree	Undecided	Disagree		Agree	Agree	Agree	Agree	NSAIDs	Celecoxib 400 mg	Continue NSAIDS and weight loss therapy	NSAIDS	Tylenol		Opioid
PGY1	No	Neutral	Never received any formal training	California, New York, Pennsylvania	75,000	45,000	TRUE	Prescription Drug Monitoring Program	Anytime an opioid prescription is given.	Agree	Strongly disagree	Disagree	Strongly agree	Undecided	Disagree		Strongly agree	Strongly agree	Strongly agree	Strongly agree	NSAIDs	Naratriptan 2.5 mg	0-10 tablets of 5mg Tramadol	0-10 tablets of 5mg Oxycodone	NSAIDs	NSAIDs	Opioid
PGY1	No	Unsatisfied	Medical School	Pennsylvania, West Virginia	45,000	25,000	TRUE	Prescription Drug Monitoring Program	Anytime an opioid prescription is given.	Disagree	Strongly disagree	Disagree	Agree	Agree	Agree		Agree	Agree	Agree	Strongly agree	Duloxetine	Naratriptan 2.5 mg	Exercise	0-10 tablets of 5mg Oxycodone	Tylenol	NSAIDs	Opioid
PGY3	No	Unsatisfied	Residency, Personal reading	Alabama	45,000	25,000	FALSE	Prescription Drug Monitoring Program	Anytime an opioid prescription is given.	Agree	Strongly disagree	Disagree	Undecided	Disagree	Agree		Agree	Agree	Agree	Agree	NSAIDs	Naratriptan 2.5 mg	Acetaminophen	0-10 tablets of 5mg Oxycodone	Tylenol	Tylenol	Opioid
PGY2	No	Neutral	Medical School, Residency, Personal reading	Alabama, Kentucky, Pennsylvania	45,000	45,000	TRUE	Prescription Drug Monitoring Program	Anytime an opioid prescription is given.	Agree		Agree	Undecided	Agree	Disagree		Agree	Strongly agree	Agree	Agree	NSAIDs	Codeine/acetaminophen 25/400 mg	Continue NSAIDS and weight loss therapy	0-10 tablets of 5mg Oxycodone	Tylenol	NSAIDs	Opioid
PGY1	No	Unsatisfied	Residency	New York, Pennsylvania, West Virginia	45,000	45,000	TRUE	Prescription Drug Monitoring Program	Anytime an opioid prescription is given.	Agree	Strongly disagree	Strongly disagree	Agree	Disagree	Disagree		Strongly agree	Agree	Agree	Strongly agree	NSAIDs	Chlorpromazine IV 12.5 mg	Acetaminophen	0-10 tablets of 5mg Oxycodone	Tylenol	NSAIDs	Opioid
PGY1	No	Neutral	Medical School, Residency	California, New York, Pennsylvania	75,000	25,000	TRUE	Prescription Drug Monitoring Program	Anytime an opioid prescription is given.	Agree	Strongly disagree	Undecided	Undecided	Disagree	Disagree		Agree	Agree	Disagree	Strongly agree	NSAIDs	Naratriptan 2.5 mg	0-10 tablets of 5mg Oxycodone	NSAIDS	Tylenol	Tylenol	Tylenol
PGY3	Yes	Neutral	Medical School, Residency, Personal reading	Kentucky, Delaware, West Virginia	75,000	45,000	FALSE	Prescription Drug Monitoring Program	Anytime an opioid prescription is given.	Agree	Disagree	Undecided	Strongly agree	Agree	Disagree		Strongly agree	Disagree	Disagree	Agree	NSAIDs	Naratriptan 2.5 mg	Continue NSAIDS and weight loss therapy	NSAIDS	Tylenol	NSAIDs	Opioid
PGY1	No	Unsatisfied	Residency, Personal reading	Kentucky, Pennsylvania, West Virginia	75,000	45,000	FALSE	Prescription Drug Monitoring Program	Anytime an opioid prescription is given.	Undecided	Strongly disagree	Disagree	Strongly disagree	Undecided	Undecided		Agree	Agree	Disagree	Agree	NSAIDs	Chlorpromazine IV 12.5 mg	Acetaminophen	0-10 tablets of 5mg Oxycodone	Tylenol	Tylenol	Tylenol
PGY1	No	Neutral	Medical School, Residency	Kentucky, Pennsylvania, West Virginia	75,000	45,000	TRUE	Prescription Drug Monitoring Program	Anytime an opioid prescription is given.	Agree	Strongly disagree	Undecided	Strongly agree	Undecided	Disagree		Undecided	Strongly agree	Strongly agree	Agree	NSAIDs	Naratriptan 2.5 mg	0-10 tablets of 5mg Tramadol	NSAIDS	Tylenol	NSAIDs	Opioid
PGY1	No	Unsatisfied	Residency		45,000	25,000	TRUE	Prescription Drug Monitoring Program	Anytime an opioid prescription is given.	Undecided	Strongly disagree	Disagree	Agree	Disagree	Disagree		Agree	Agree	Undecided	Strongly agree	NSAIDs	Naratriptan 2.5 mg	Continue NSAIDS and weight loss therapy	NSAIDS	Tylenol	Tylenol	Tylenol
PGY2	No	Unsatisfied	Medical School, Residency	Alabama, Kentucky, New York	45,000	45,000	TRUE	Prescription Drug Monitoring Program	Anytime an opioid prescription is given.	Agree	Strongly disagree	Agree	Agree	Disagree	Disagree		Agree	Agree	Agree	Strongly agree	NSAIDs	Celecoxib 400 mg	0-10 tablets of 5mg Tramadol	0-10 tablets of 5mg Oxycodone	Tylenol	Tylenol	Opioid
PGY2	No	Neutral	Medical School, Residency, Personal reading	Alabama, Pennsylvania, West Virginia	75,000	45,000	TRUE	Prescription Drug Monitoring Program	Anytime an opioid prescription is given.	Agree	Disagree	Disagree	Agree	Disagree	Disagree		Agree	Agree	Disagree	Agree	NSAIDs	Chlorpromazine IV 12.5 mg	Exercise	0-10 tablets of 5mg Oxycodone	Tylenol	NSAIDs	Opioid
PGY2	No	Unsatisfied	Medical School, Personal reading	Kentucky, Pennsylvania, West Virginia	75,000	15,000	TRUE	Prescription Drug Monitoring Program	Anytime an opioid prescription is given.	Agree	Disagree	Agree	Undecided	Agree	Agree		Strongly agree	Strongly agree	Strongly agree	Strongly agree	NSAIDs	Naratriptan 2.5 mg	Acetaminophen	NSAIDS	Tylenol	NSAIDs	Opioid
PGY3	Yes	Unsatisfied	Medical School, Residency, Personal reading	Kentucky, New York, Pennsylvania	45,000	15,000	TRUE	Prescription Drug Monitoring Program	Anytime an opioid prescription is given.	Disagree	Strongly disagree	Disagree	Disagree	Agree	Agree		Agree	Agree	Undecided	Undecided	NSAIDs	Chlorpromazine IV 12.5 mg	Continue NSAIDS and weight loss therapy	NSAIDS	Tylenol	NSAIDs	Opioid
PGY1	No	Unsatisfied	Medical School	New York, Pennsylvania, West Virginia	45,000	25,000	TRUE	Prescription Drug Monitoring Program	Anytime an opioid prescription is given.	Agree	Strongly disagree	Disagree	Undecided	Disagree	Disagree		Agree	Agree	Agree	Agree	NSAIDs	Celecoxib 400 mg	0-10 tablets of 5mg Tramadol	NSAIDS	Tylenol	Tylenol	Tylenol
PGY2	No	Unsatisfied	Residency, Personal reading	Alabama, Kentucky, New York	45,000	25,000	TRUE	Prescription Drug Monitoring Program	Anytime an opioid prescription is given.	Agree	Strongly disagree	Undecided	Agree	Strongly disagree	Strongly disagree		Agree	Agree	Agree	Agree	NSAIDs	Naratriptan 2.5 mg	Acetaminophen	0-10 tablets of 5mg Oxycodone	NSAIDs	Tylenol	Tylenol

**Table 11:** IM resident post-test data PGY: post-graduate year; DEA: Drug Enforcement Administration; PDMP: prescription drug monitoring program; PA: Pennsylvania; NSAID: non-steroidal anti-inflammatory drug; IM: internal medicine

1. Please select your current level of training:	2. Do you hold a DEA License?	3. How satisfied are you with your current level of opioid-prescribing training?	4. When did you receive your opioid-prescribing training? (Select all that apply)	5. Which three states have the highest percentage of opioid-related deaths per capita:(circle 3 states)	6. In 2017, how many drug overdose deaths were due to opioids?	7. In 2017, how many deaths were a result of heroin overdose?	8. Nearly half of all opioid related overdoses are due to valid prescription opioids.	9. What is the PDMP?	10. How often should the PDMP be referenced?	11. Opioids are effective in pain management.	12. Every patient that presents to the office with pain should receive opioids.	13. I feel comfortable in my knowledge of non-opioid pain management.	14. If I suspect someone is abusing opioids, I do not prescribe opioids to them.	15. Patient gender may affect my judgement of a patient's pain intensity	16. Patient race may affect my judgement of a patient's pain intensity	17. If a patient presents to the ED repeatedly asking for more pain medication, this could be due to a missed diagnosis of the underlying pain source.	18. I ask my patients about the severity of their pain.	19. I include patient-reported pain levels in my notes.	20. I think that proper pain management is associated with better patient outcomes.	21. For an adult presenting with chronic low back pain, I would initially prescribe: (circle only one)	22. A 25-year-old female presents to the office with an acute episodic migraine According to the American Headache Society 2015 Guidelines, what treatment has Level A evidence?	23. A 65-year-old man returns to the clinic for joint pain in his knees. He has a history of osteoarthritis and states that it is difficult for him to complete daily tasks. His pain was not treated by NSAIDs or weight loss. What should be the next line of treatment?	24. A 35-year-old male presents to the office with nephrolithiasis. His eGFR is >90ml/min and he has no history of GI bleed. How would you initially treat his pain?	25. For patients experiencing mild pain, I usually initially prescribe (circle one)	26. For patients experiencing moderate pain, I usually initially prescribe (circle one)	27. For patients experiencing severe pain, I usually initially prescribe (circle only one)
PGY1	No	Unsatisfied	Medical School, Residency, Personal reading	Alabama, Kentucky, Pennsylvania, West Virginia	25,000	15,000	TRUE	Prescription Drug Monitoring Program	Anytime an opioid prescription is given.	Agree	Strongly disagree	Undecided	Agree	Agree	Agree	Agree	Agree	Agree	Strongly agree	NSAIDs	Naratriptan 2.5 mg	0-10 tablets of 5mg Oxycodone	NSAIDS	Tylenol	NSAIDs	Opioid
PGY2	No	Neutral	Medical School, Residency	Kentucky, Pennsylvania, West Virginia	45,000	15,000	TRUE	Prescription Drug Monitoring Program	Anytime an opioid prescription is given.	Agree	Strongly disagree	Undecided	Strongly agree	Disagree	Disagree	Agree	Agree	Agree	Agree	NSAIDs	Chlorpromazine IV 12.5 mg	Acetaminophen	0-10 tablets of 5mg Oxycodone	Tylenol	Tylenol	Opioid
PGY2	No	Neutral	Medical School, Residency	Kentucky, Pennsylvania, West Virginia	75,000	15,000	TRUE	Prescription Drug Monitoring Program	Anytime an opioid prescription is given.	Strongly agree	Strongly disagree	Agree	Undecided	Strongly disagree	Strongly disagree	Agree	Agree	Agree	Agree	NSAIDs	Naratriptan 2.5 mg	0-10 tablets of 5mg Tramadol	NSAIDS	NSAIDs	NSAIDs	Tylenol
PGY1	No	Unsatisfied	Medical School	Delaware, South Carolina, West Virginia	45,000	15,000	TRUE	Prescription Drug Monitoring Program	Anytime an opioid prescription is given.	Strongly agree	Strongly disagree	Disagree	Agree	Agree	Agree	Agree	Strongly agree	Strongly agree	Agree	NSAIDs	Naratriptan 2.5 mg	0-10 tablets of 5mg Tramadol	NSAIDS	Tylenol	NSAIDs	Opioid
PGY3	No	Neutral	Medical School, Residency	Alabama, Kentucky, Pennsylvania, West Virginia	75,000	15,000	TRUE	Prescription Drug Monitoring Program	Anytime an opioid prescription is given.	Undecided	Strongly disagree	Agree	Strongly agree	Strongly disagree	Strongly disagree	Agree	Agree	Disagree	Agree	NSAIDs	Naratriptan 2.5 mg	0-10 tablets of 5mg Tramadol	NSAIDS	NSAIDs	NSAIDs	Opioid
PGY2	No	Neutral	Medical School, Residency	Kentucky, Pennsylvania, West Virginia	45,000	15,000	TRUE	Prescription Drug Monitoring Program	Anytime an opioid prescription is given.	Agree	Strongly disagree	Undecided	Strongly agree	Disagree	Disagree	Agree	Agree	Agree	Agree	NSAIDs	Chlorpromazine IV 12.5 mg	Acetaminophen	0-10 tablets of 5mg Oxycodone	Tylenol	Tylenol	Opioid
PGY1	No	Neutral	Medical School	Kentucky, Delaware, West Virginia	25,000	15,000	TRUE	Prescription Drug Monitoring Program	Anytime an opioid prescription is given.	Disagree	Strongly disagree	Disagree	Strongly agree	Disagree	Agree	Agree	Strongly agree	Agree	Agree	NSAIDs	Naratriptan 2.5 mg	0-10 tablets of 5mg Tramadol	NSAIDS	Tylenol	NSAIDs	Opioid
PGY1	No	Unsatisfied	Medical School	Delaware, Pennsylvania, West Virginia	45,000	15,000	TRUE	Prescription Drug Monitoring Program	Anytime an opioid prescription is given.	Agree	Strongly disagree	Disagree	Agree	Disagree	Disagree	Agree	Agree	Agree	Agree	Tramadol	Naratriptan 2.5 mg	Acetaminophen	No pain medication	Tylenol	Tylenol	Tylenol
PGY3	No	Neutral	Residency	Delaware, Pennsylvania, West Virginia	75,000	15,000	TRUE	Prescription Drug Monitoring Program	Anytime an opioid prescription is given.	Agree	Strongly disagree	Agree	Agree	Undecided	Undecided	Agree	Agree	Agree	Strongly agree	NSAIDs	Naratriptan 2.5 mg	Exercise	NSAIDS	Tylenol	NSAIDs	Opioid
PGY2	No	Unsatisfied	Residency	Delaware, Pennsylvania, West Virginia	45,000	15,000	TRUE	Prescription Drug Monitoring Program	Anytime an opioid prescription is given.	Agree	Strongly disagree	Agree	Agree	Agree	Agree	Agree	Agree	Agree	Strongly agree	NSAIDs	Naratriptan 2.5 mg	0-10 tablets of 5mg Tramadol	NSAIDS	NSAIDs	NSAIDs	NSAIDs
PGY2	No	Unsatisfied	Residency	California, New York, Pennsylvania	45,000	25,000	TRUE	Prescription Drug Monitoring Program	Anytime an opioid prescription is given.	Strongly agree	Strongly disagree	Agree	Strongly agree	Strongly agree	Undecided	Agree	Strongly agree	Agree	Strongly agree	NSAIDs	Celecoxib 400 mg	Acetaminophen	NSAIDS	Tylenol	Tylenol	Opioid
PGY2	No	Unsatisfied	Residency	California, New York, Pennsylvania	45,000	25,000	TRUE	Prescription Drug Monitoring Program	Anytime an opioid prescription is given.	Strongly agree	Strongly disagree	Agree	Strongly agree	Strongly agree	Undecided	Agree	Strongly agree	Agree	Strongly agree	NSAIDs	Celecoxib 400 mg	Acetaminophen	NSAIDS	Tylenol	Tylenol	Opioid
PGY2	No	Neutral	Medical School, Residency	Kentucky, Pennsylvania, West Virginia	45,000	15,000	TRUE	Prescription Drug Monitoring Program	Anytime an opioid prescription is given.	Agree	Strongly disagree	Undecided	Strongly agree	Disagree	Disagree	Agree	Agree	Agree	Agree	NSAIDs	Chlorpromazine IV 12.5 mg	Acetaminophen	0-10 tablets of 5mg Oxycodone	Tylenol	Tylenol	Opioid
PGY1	No	Unsatisfied	Never received any formal training	Kentucky, New York, Pennsylvania	45,000	15,000	TRUE	Prescription Drug Monitoring Program	Anytime an opioid prescription is given.	Strongly agree	Strongly disagree	Disagree	Undecided	Disagree	Disagree	Agree	Agree	Agree	Agree	NSAIDs	Naratriptan 2.5 mg	Exercise	NSAIDS	Tylenol	Tylenol	NSAIDs
PGY1	No	Satisfied	Medical School, Residency	California, Delaware, West Virginia	45,000	15,000	TRUE	Prescription Drug Monitoring Program	Anytime an opioid prescription is given.	Agree	Strongly disagree	Agree	Agree	Disagree	Disagree	Agree	Agree	Agree	Agree	NSAIDs	Naratriptan 2.5 mg	0-10 tablets of 5mg Tramadol	NSAIDS	Tylenol	NSAIDs	Opioid
PGY3	No	Satisfied	Never received any formal training	Alabama, Kentucky, West Virginia	45,000	45,000	TRUE	Prescription Drug Monitoring Program	Anytime an opioid prescription is given.	Agree	Strongly disagree	Agree	Agree	Disagree	Strongly disagree	Agree	Agree	Agree	Agree	NSAIDs	Naratriptan 2.5 mg	Continue NSAIDS and weight loss therapy	0-10 tablets of 5mg Oxycodone	Tylenol	Tylenol	Opioid
PGY1	No	Neutral	Medical School, Residency	Alabama, Kentucky, West Virginia	45,000	15,000	TRUE	Prescription Drug Monitoring Program	Anytime an opioid prescription is given.	Agree	Strongly disagree	Agree	Strongly agree	Undecided	Agree	Agree	Strongly agree	Strongly agree	Strongly agree	NSAIDs	Naratriptan 2.5 mg	0-10 tablets of 5mg Tramadol	0-10 tablets of 5mg Oxycodone	Tylenol	NSAIDs	Opioid
PGY1	No	Neutral	Medical School	Kentucky, Pennsylvania, West Virginia	25,000	45,000	TRUE	Prescription Drug Monitoring Program	Anytime an opioid prescription is given.	Strongly agree	Strongly disagree	Agree	Agree	Agree	Disagree	Agree	Agree	Agree	Agree	NSAIDs	Naratriptan 2.5 mg	0-10 tablets of 5mg Tramadol	NSAIDS	Tylenol	NSAIDs	Opioid
PGY1	No	Unsatisfied	Never received any formal training	Delaware, Pennsylvania, West Virginia	45,000	15,000	TRUE	Prescription Drug Monitoring Program	Anytime an opioid prescription is given.	Agree	Strongly disagree	Undecided	Strongly agree	Undecided	Undecided	Agree	Agree	Agree	Agree	NSAIDs	Chlorpromazine IV 12.5 mg	Acetaminophen	NSAIDS	Tylenol	NSAIDs	NSAIDs
PGY2	No	Unsatisfied	Residency, Personal reading	Delaware, Pennsylvania, West Virginia	75,000	15,000	TRUE	Prescription Drug Monitoring Program	Anytime an opioid prescription is given.	Agree	Strongly disagree	Agree	Agree	Disagree	Disagree	Agree	Agree	Agree	Agree	NSAIDs	Naratriptan 2.5 mg	0-10 tablets of 5mg Tramadol	NSAIDS	NSAIDs	Tylenol	Tylenol

**Table 12:** GS pre-test data PGY: post-graduate year; DEA: Drug Enforcement Administration; PDMP: prescription drug monitoring program; PA: Pennsylvania; NSAID: non-steroidal anti-inflammatory drug; GS: general surgery

1. Please select your current level of training:	2. Do you hold a DEA License?	3. How satisfied are you with your current level of opioid-prescribing training?	4. When did you receive your opioid-prescribing training? (Select all that apply)	5. Which three states have the highest percentage of opioid-related deaths per capita:(circle 3 states)	6. In 2017, how many drug overdose deaths were due to opioids?	7. In 2017, how many deaths were a result of heroin overdose?	8. Nearly half of all opioid related overdoses are due to valid prescription opioids.	9. What is the PDMP?	10. How often should the PDMP be referenced?	11. Opioids are effective in pain management.	12. Every patient should receive opioids following surgery.	13. I feel comfortable in my knowledge of non-opioid pain management.	14. If I suspect someone is abusing opioids, I do not prescribe opioids to them.	15. Patient gender may affect my judgement of a patient's pain intensity	16. Patient race may affect my judgement of a patient's pain intensity	17. I ask my patients about the severity of their pain.	18. I include patient-reported pain levels in my notes.	19. For a patient being discharged home after an open appendectomy, I would typically prescribe: (circle only one)	20. For a patient being discharged home after a sleeve gastrectomy, I would typically prescribe: (circle only one)	21. For a patient being discharged home after a laparoscopic cholecystectomy, I would typically prescribe: (circle only one)	22. For a patient being discharged home after a laparoscopic Nissen fundoplication, I would typically prescribe: (circle only one)	23. For a patient being discharged home after an open small bowel resection, I would typically prescribe: (circle only one)	24. For a patient being discharged home after an open colectomy, I would typically prescribe: (circle only one)	25. For a patient being discharged home after a major hernia repair, I would typically prescribe: (circle only one)	26. For patients experiencing mild pain, I usually initially prescribe (circle one)	27. For patients experiencing moderate pain, I usually initially prescribe (circle one)	28. For patients experiencing severe pain, I usually initially prescribe (circle only one)
PGY4	Yes	Satisfied	Medical School, Residency, Personal reading	Kentucky, New York, South Carolina	45,000	25,000	TRUE	Prescribing Directory of Medical Providers	Anytime an opioid prescription is given	Agree	Disagree	Disagree	Disagree	Undecided	Disagree	Agree	Disagree	0-10 tablets of 5mg Oxycodone	11-20 tablets of 5mg Oxycodone	0-10 tablets of 5mg Oxycodone	0-10 tablets of 5mg Oxycodone	0-10 tablets of 5mg Oxycodone	0-10 tablets of 5mg Oxycodone	0-15 tablets of 5mg Oxycodone	Tylenol	Opioid	Opioid
PGY3	Yes	Very satisfied	Medical School, Residency	California, New York, Pennsylvania	75,000	45,000	TRUE	Prescription Drug Monitoring Program	Anytime an opioid prescription is given	Strongly agree	Disagree	Strongly agree	Disagree	Disagree	Disagree	Strongly agree	Disagree	0-10 tablets of 5mg Oxycodone	11-20 tablets of 5mg Oxycodone	0-10 tablets of 5mg Oxycodone	0-15 tablets of 5mg Oxycodone	0-25 tablets of 5mg Oxycodone	0-25 tablets of 5mg Oxycodone	0-25 tablets of 5mg Oxycodone	Tylenol	Tylenol	Opioid
PGY4	Yes	Very satisfied	Medical School, Residency, Personal reading	New York, Pennsylvania, West Virginia	75,000	45,000	TRUE	Prescription Drug Monitoring Program	Anytime an opioid prescription is given	Agree	Strongly disagree	Strongly agree	Strongly agree	Strongly disagree	Strongly disagree	Strongly agree	Strongly agree	0-10 tablets of 5mg Oxycodone	11-20 tablets of 5mg Oxycodone	0-5 tablets of 5mg Oxycodone	0-15 tablets of 5mg Oxycodone	0-15 tablets of 5mg Oxycodone	0-20 tablets of 5mg Oxycodone	0-20 tablets of 5mg Oxycodone	Tylenol	Opioid	Opioid
PGY2	Yes	Neutral	Residency	California, New York, Pennsylvania	25,000	15,000	FALSE	Prescription Drug Monitoring Program	Anytime an opioid prescription is given	Agree	Disagree	Agree	Agree	Strongly disagree	Strongly disagree	Agree	Agree	0-10 tablets of 5mg Oxycodone	0-10 tablets of 5mg Oxycodone	0-10 tablets of 5mg Oxycodone	0-15 tablets of 5mg Oxycodone	0-15 tablets of 5mg Oxycodone	0-15 tablets of 5mg Oxycodone	0-15 tablets of 5mg Oxycodone	Tylenol	Tylenol	Opioid
PGY4	Yes	Neutral	Residency	Alabama, California, New York	45,000	45,000	TRUE	Prescription Drug Monitoring Program	Anytime an opioid prescription is given	Agree	Strongly disagree	Agree	Undecided	Agree	Agree	Strongly agree	Undecided	0-10 tablets of 5mg Oxycodone	0-10 tablets of 5mg Oxycodone	0-10 tablets of 5mg Oxycodone	0-10 tablets of 5mg Oxycodone	0-15 tablets of 5mg Oxycodone	0-15 tablets of 5mg Oxycodone	0-15 tablets of 5mg Oxycodone	Tylenol	Opioid	Opioid
PGY4	Yes	Neutral	Residency	Alabama, Kentucky, West Virginia	75,000	75,000	TRUE	Prescription Drug Monitoring Program	Anytime an opioid prescription is given	Agree	Disagree	Strongly disagree	Disagree	Undecided	Undecided	Agree	Undecided	Only non-opioid pain medications	Only non-opioid pain medications	0-5 tablets of 5mg Oxycodone	0-5 tablets of 5mg Oxycodone	0-5 tablets of 5mg Oxycodone	0-5 tablets of 5mg Oxycodone	0-5 tablets of 5mg Oxycodone	NSAIDs	Tylenol	Tylenol
PGY1	No	Satisfied	Medical School, Residency	Alabama, South Carolina, West Virginia	25,000	25,000	TRUE	Prescription Drug Monitoring Program	Anytime an opioid prescription is given	Strongly agree	Undecided	Undecided	Disagree	Agree	Disagree	Strongly agree	Agree	11-20 tablets of 5mg Oxycodone	11-20 tablets of 5mg Oxycodone	0-15 tablets of 5mg Oxycodone	0-15 tablets of 5mg Oxycodone	0-20 tablets of 5mg Oxycodone	0-20 tablets of 5mg Oxycodone	0-15 tablets of 5mg Oxycodone	Tylenol	Tylenol	Tylenol
PGY3	Yes	Very satisfied	Medical School, Residency	Alabama, Pennsylvania, West Virginia	75,000	45,000	TRUE	Prescription Drug Monitoring Program	Anytime an opioid prescription is given	Strongly agree	Strongly disagree	Strongly agree	Undecided	Strongly disagree	Strongly disagree	Strongly agree	Agree	0-10 tablets of 5mg Oxycodone	0-10 tablets of 5mg Oxycodone	0-10 tablets of 5mg Oxycodone	0-10 tablets of 5mg Oxycodone	0-10 tablets of 5mg Oxycodone	0-10 tablets of 5mg Oxycodone	0-20 tablets of 5mg Oxycodone	Tylenol	Tylenol	Tylenol
PGY2	No	Satisfied	Medical School, Residency, Personal reading	New York, Pennsylvania, South Carolina	45,000	75,000	TRUE	Prescription Drug Monitoring Program	Anytime an opioid prescription is given	Agree	Disagree	Agree	Undecided	Strongly disagree	Strongly disagree	Agree	Agree	0-10 tablets of 5mg Oxycodone	0-10 tablets of 5mg Oxycodone	0-10 tablets of 5mg Oxycodone	0-10 tablets of 5mg Oxycodone	0-10 tablets of 5mg Oxycodone	0-10 tablets of 5mg Oxycodone	0-10 tablets of 5mg Oxycodone	Tylenol	NSAIDs	Opioid
PGY4	Yes	Satisfied	Residency	California, New York, Pennsylvania	45,000	45,000	TRUE	Prescription Drug Monitoring Program	Anytime an opioid prescription is given	Agree	Disagree	Agree	Agree	Agree	Agree	Agree	Agree	0-10 tablets of 5mg Oxycodone	0-10 tablets of 5mg Oxycodone	0-5 tablets of 5mg Oxycodone	0-5 tablets of 5mg Oxycodone	0-10 tablets of 5mg Oxycodone	0-10 tablets of 5mg Oxycodone	0-10 tablets of 5mg Oxycodone	Tylenol	Opioid	Opioid
PGY1	Yes	Neutral	Residency	California, New York, Pennsylvania	25,000	15,000	TRUE	Prescription Drug Monitoring Program	Anytime an opioid prescription is given	Agree	Disagree	Agree	Disagree	Agree	Agree	Agree	Disagree	0-10 tablets of 5mg Oxycodone	21-30 tablets of 5mg Oxycodone	0-10 tablets of 5mg Oxycodone	0-10 tablets of 5mg Oxycodone	0-15 tablets of 5mg Oxycodone	0-15 tablets of 5mg Oxycodone	0-5 tablets of 5mg Oxycodone	Tylenol	Opioid	Opioid
PGY5	Yes	Satisfied	Medical School, Residency, Personal reading	Kentucky, New York, Pennsylvania	45,000	75,000	TRUE	Prescription Drug Monitoring Program	Anytime an opioid prescription is given	Agree	Disagree	Agree	Undecided	Strongly disagree	Strongly disagree	Agree	Agree	0-10 tablets of 5mg Oxycodone	11-20 tablets of 5mg Oxycodone	0-10 tablets of 5mg Oxycodone	0-15 tablets of 5mg Oxycodone	0-20 tablets of 5mg Oxycodone	0-20 tablets of 5mg Oxycodone	0-20 tablets of 5mg Oxycodone	Tylenol	Opioid	Opioid
PGY3	Yes	Unsatisfied	Medical School, Residency, Personal reading	California, New York, Pennsylvania	45,000	25,000	TRUE	Prescription Drug Monitoring Program	Anytime an opioid prescription is given	Disagree	Strongly disagree	Undecided	Agree	Strongly disagree	Strongly disagree	Strongly agree	Strongly agree	Only non-opioid pain medications	0-10 tablets of 5mg Oxycodone	0-5 tablets of 5mg Oxycodone	0-5 tablets of 5mg Oxycodone	0-10 tablets of 5mg Oxycodone	0-10 tablets of 5mg Oxycodone	0-10 tablets of 5mg Oxycodone	Tylenol	NSAIDs	Opioid
PGY5	Yes	Satisfied	Medical School, Residency	Alabama, California, New York	45,000	25,000	TRUE	Prescription Drug Monitoring Program	Anytime an opioid prescription is given	Agree	Disagree	Strongly agree	Agree	Disagree	Strongly disagree	Agree	Disagree	0-10 tablets of 5mg Oxycodone	0-10 tablets of 5mg Oxycodone	0-10 tablets of 5mg Oxycodone	0-15 tablets of 5mg Oxycodone	0-10 tablets of 5mg Oxycodone	0-15 tablets of 5mg Oxycodone	0-20 tablets of 5mg Oxycodone	Tylenol	NSAIDs	Opioid
PGY5	Yes	Satisfied	Residency	Kentucky, Delaware, West Virginia	75,000	45,000	TRUE	Prescription Drug Monitoring Program	Anytime an opioid prescription is given	Agree	Disagree	Agree	Agree	Disagree	Strongly disagree	Agree	Agree	0-10 tablets of 5mg Oxycodone	0-10 tablets of 5mg Oxycodone	0-5 tablets of 5mg Oxycodone	0-10 tablets of 5mg Oxycodone	0-20 tablets of 5mg Oxycodone	0-20 tablets of 5mg Oxycodone	0-25 tablets of 5mg Oxycodone	Tylenol	NSAIDs	Opioid
PGY5	Yes	Satisfied	Residency	California, Delaware, Pennsylvania	75,000	15,000	TRUE	Prescription Drug Monitoring Program	Anytime an opioid prescription is given	Agree	Disagree	Agree	Agree	Disagree	Disagree	Agree	Agree	0-10 tablets of 5mg Oxycodone	0-10 tablets of 5mg Oxycodone	0-10 tablets of 5mg Oxycodone	0-5 tablets of 5mg Oxycodone	0-15 tablets of 5mg Oxycodone	0-15 tablets of 5mg Oxycodone	0-10 tablets of 5mg Oxycodone	NSAIDs	NSAIDs	Opioid
PGY2	Yes	Satisfied	Residency	California, New York, West Virginia	75,000	75,000	TRUE	Prescribing Directory of Medical Providers	Anytime an opioid prescription is given	Agree	Disagree	Agree	Agree	Disagree	Disagree	Agree	Agree	Only non-opioid pain medications	Only non-opioid pain medications	0-5 tablets of 5mg Oxycodone	0-5 tablets of 5mg Oxycodone	0-15 tablets of 5mg Oxycodone	0-15 tablets of 5mg Oxycodone	0-20 tablets of 5mg Oxycodone	Tylenol	NSAIDs	Opioid

**Table 13:** GS post-test data PGY: post-graduate year; DEA: Drug Enforcement Administration; PDMP: prescription drug monitoring program; PA: Pennsylvania; NSAID: non-steroidal anti-inflammatory drug; GS: general surgery

1. Please select your current level of training:	2. Do you hold a DEA License?	3. How satisfied are you with your current level of opioid-prescribing training?	4. When did you receive your opioid-prescribing training? (Select all that apply)	5. Which three states have the highest percentage of opioid-related deaths per capita:(circle 3 states)	6. In 2017, how many drug overdose deaths were due to opioids?	7. In 2017, how many deaths were a result of heroin overdose?	8. Nearly half of all opioid related overdoses are due to valid prescription opioids.	9. What is the PDMP?	10. How often should the PDMP be referenced?	11. Opioids are effective in pain management.	12. Every patient should receive opioids following surgery.	13. I feel comfortable in my knowledge of non-opioid pain management.	14. If I suspect someone is abusing opioids, I do not prescribe opioids to them.	15. Patient gender may affect my judgement of a patient's pain intensity	16. Patient race may affect my judgement of a patient's pain intensity	17. I ask my patients about the severity of their pain.	18. I include patient-reported pain levels in my notes.	19. For a patient being discharged home after an open appendectomy, I would typically prescribe: (circle only one)	20. For a patient being discharged home after a sleeve gastrectomy, I would typically prescribe: (circle only one)	21. For a patient being discharged home after a laparoscopic cholecystectomy, I would typically prescribe: (circle only one)	22. For a patient being discharged home after a laparoscopic Nissen fundoplication, I would typically prescribe: (circle only one)	23. For a patient being discharged home after an open small bowel resection, I would typically prescribe: (circle only one)	24. For a patient being discharged home after an open colectomy, I would typically prescribe: (circle only one)	25. For a patient being discharged home after a major hernia repair, I would typically prescribe: (circle only one)	26. For patients experiencing mild pain, I usually initially prescribe (circle one)	27. For patients experiencing moderate pain, I usually initially prescribe (circle one)	28. For patients experiencing severe pain, I usually initially prescribe (circle only one)
PGY2	Yes	Satisfied	Medical School, Residency, Personal reading	Delaware, Pennsylvania, West Virginia	75,000	75,000	TRUE	Prescription Drug Monitoring Program	Anytime an opioid prescription is given	Agree	Agree	Agree	Disagree	Strongly disagree	Strongly disagree	Agree	Agree	0-10 tablets of 5mg Oxycodone	0-10 tablets of 5mg Oxycodone	0-15 tablets of 5mg Oxycodone	0-10 tablets of 5mg Oxycodone	0-10 tablets of 5mg Oxycodone	0-15 tablets of 5mg Oxycodone	0-10 tablets of 5mg Oxycodone	Tylenol	NSAIDs	Opioid
PGY5	Yes	Satisfied	Medical School, Residency, Personal reading	Delaware, Pennsylvania, West Virginia	45,000	15,000	TRUE	Prescription Drug Monitoring Program	Anytime an opioid prescription is given	Agree	Disagree	Agree	Agree	Strongly disagree	Strongly disagree	Strongly agree	Agree	0-10 tablets of 5mg Oxycodone	0-10 tablets of 5mg Oxycodone	0-15 tablets of 5mg Oxycodone	0-10 tablets of 5mg Oxycodone	0-15 tablets of 5mg Oxycodone	0-15 tablets of 5mg Oxycodone	0-10 tablets of 5mg Oxycodone	Tylenol	NSAIDs	Opioid
PGY3	Yes	Very satisfied	Medical School, Residency	Delaware, Pennsylvania, West Virginia	45,000	15,000	TRUE	Prescription Drug Monitoring Program	Anytime an opioid prescription is given	Strongly agree	Disagree	Strongly agree	Disagree	Disagree	Disagree	Strongly agree	Disagree	0-10 tablets of 5mg Oxycodone	0-10 tablets of 5mg Oxycodone	0-15 tablets of 5mg Oxycodone	0-10 tablets of 5mg Oxycodone	0-15 tablets of 5mg Oxycodone	0-15 tablets of 5mg Oxycodone	0-10 tablets of 5mg Oxycodone	Tylenol	Tylenol	Opioid
PGY4	Yes	Very satisfied	Medical School, Residency	Delaware, Pennsylvania, West Virginia	45,000	15,000	TRUE	Prescription Drug Monitoring Program	Anytime an opioid prescription is given	Agree	Strongly disagree	Strongly agree	Agree	Strongly disagree	Strongly disagree	Strongly agree	Strongly agree	0-10 tablets of 5mg Oxycodone	0-10 tablets of 5mg Oxycodone	0-10 tablets of 5mg Oxycodone	0-10 tablets of 5mg Oxycodone	0-15 tablets of 5mg Oxycodone	0-20 tablets of 5mg Oxycodone	0-15 tablets of 5mg Oxycodone	Tylenol	Opioid	Opioid
PGY3	Yes	Very satisfied	Medical School, Residency	Delaware, Pennsylvania, West Virginia	75,000	25,000	TRUE	Prescription Drug Monitoring Program	Anytime an opioid prescription is given	Strongly agree	Strongly disagree	Strongly agree	Agree	Strongly disagree	Strongly disagree	Strongly agree	Strongly agree	0-10 tablets of 5mg Oxycodone	0-10 tablets of 5mg Oxycodone	0-10 tablets of 5mg Oxycodone	0-15 tablets of 5mg Oxycodone	0-10 tablets of 5mg Oxycodone	0-10 tablets of 5mg Oxycodone	0-15 tablets of 5mg Oxycodone	Tylenol	Tylenol	Tylenol
PGY4	Yes	Very satisfied	Residency	Delaware, Pennsylvania, West Virginia	45,000	15,000	TRUE	Prescription Drug Monitoring Program	Anytime an opioid prescription is given	Agree	Strongly disagree	Agree	Undecided	Strongly disagree	Strongly disagree	Strongly agree	Agree	0-10 tablets of 5mg Oxycodone	0-10 tablets of 5mg Oxycodone	0-15 tablets of 5mg Oxycodone	0-10 tablets of 5mg Oxycodone	0-10 tablets of 5mg Oxycodone	0-10 tablets of 5mg Oxycodone	0-10 tablets of 5mg Oxycodone	Tylenol	Opioid	Opioid
PGY2	Yes	Satisfied	Residency	Delaware, Pennsylvania, West Virginia	45,000	15,000	FALSE	Prescription Drug Monitoring Program	Anytime an opioid prescription is given	Agree	Disagree	Agree	Undecided	Strongly disagree	Strongly disagree	Agree	Agree	0-10 tablets of 5mg Oxycodone	0-10 tablets of 5mg Oxycodone	0-15 tablets of 5mg Oxycodone	0-15 tablets of 5mg Oxycodone	0-15 tablets of 5mg Oxycodone	0-15 tablets of 5mg Oxycodone	0-15 tablets of 5mg Oxycodone	Tylenol	Tylenol	Opioid
PGY4	Yes	Satisfied	Residency	Alabama, Kentucky, Pennsylvania, West Virginia	75,000	15,000	TRUE	Prescription Drug Monitoring Program	Anytime an opioid prescription is given	Agree	Strongly disagree	Agree	Disagree	Disagree	Disagree	Agree	Agree	0-10 tablets of 5mg Oxycodone	0-10 tablets of 5mg Oxycodone	0-10 tablets of 5mg Oxycodone	0-10 tablets of 5mg Oxycodone	0-10 tablets of 5mg Oxycodone	0-10 tablets of 5mg Oxycodone	0-10 tablets of 5mg Oxycodone	NSAIDs	NSAIDs	Opioid
PGY4	Yes	Satisfied	Residency	Delaware, Pennsylvania, West Virginia	45,000	15,000	TRUE	Prescription Drug Monitoring Program	Anytime an opioid prescription is given	Agree	Strongly disagree	Agree	Agree	Agree	Agree	Strongly agree	Strongly agree	0-10 tablets of 5mg Oxycodone	0-10 tablets of 5mg Oxycodone	0-15 tablets of 5mg Oxycodone	0-10 tablets of 5mg Oxycodone	0-15 tablets of 5mg Oxycodone	0-15 tablets of 5mg Oxycodone	0-15 tablets of 5mg Oxycodone	Tylenol	NSAIDs	Opioid
PGY5	Yes	Very satisfied	Medical School, Residency	Delaware, Pennsylvania, West Virginia	45,000	15,000	TRUE	Prescription Drug Monitoring Program	Anytime an opioid prescription is given	Agree	Strongly disagree	Agree	Agree	Disagree	Strongly disagree	Agree	Disagree	Only non-opioid pain medications	0-10 tablets of 5mg Oxycodone	0-10 tablets of 5mg Oxycodone	0-10 tablets of 5mg Oxycodone	0-10 tablets of 5mg Oxycodone	0-15 tablets of 5mg Oxycodone	0-10 tablets of 5mg Oxycodone	Tylenol	Opioid	Opioid
PGY2	Yes	Satisfied	Medical School, Residency, Personal reading	Delaware, Pennsylvania, West Virginia	45,000	15,000	TRUE	Prescription Drug Monitoring Program	Anytime an opioid prescription is given	Agree	Disagree	Undecided	Agree	Strongly disagree	Strongly disagree	Agree	Undecided	0-10 tablets of 5mg Oxycodone	0-10 tablets of 5mg Oxycodone	0-10 tablets of 5mg Oxycodone	0-10 tablets of 5mg Oxycodone	0-15 tablets of 5mg Oxycodone	0-15 tablets of 5mg Oxycodone	0-10 tablets of 5mg Oxycodone	Tylenol	Opioid	Opioid
PGY3	Yes	Satisfied	Medical School, Residency, Personal reading	Delaware, Pennsylvania, West Virginia	45,000	15,000	TRUE	Prescription Drug Monitoring Program	Anytime an opioid prescription is given	Agree	Disagree	Disagree	Agree	Strongly disagree	Strongly disagree	Strongly agree	Agree	0-10 tablets of 5mg Oxycodone	0-10 tablets of 5mg Oxycodone	0-10 tablets of 5mg Oxycodone	0-10 tablets of 5mg Oxycodone	0-15 tablets of 5mg Oxycodone	0-5 tablets of 5mg Oxycodone	0-10 tablets of 5mg Oxycodone	Tylenol	Opioid	Opioid
PGY5	Yes	Unsatisfied	Residency, Personal reading	Delaware, Pennsylvania, West Virginia	45,000	15,000	TRUE	Prescription Drug Monitoring Program	Anytime an opioid prescription is given	Agree	Strongly disagree	Agree	Agree	Disagree	Strongly disagree	Agree	Agree	0-10 tablets of 5mg Oxycodone	0-10 tablets of 5mg Oxycodone	0-15 tablets of 5mg Oxycodone	0-10 tablets of 5mg Oxycodone	0-15 tablets of 5mg Oxycodone	0-10 tablets of 5mg Oxycodone	0-10 tablets of 5mg Oxycodone	NSAIDs	Tylenol	Opioid
